# Diversity of Plasmids and Genes Encoding Resistance to Extended-Spectrum β-Lactamase in *Escherichia coli* from Different Animal Sources

**DOI:** 10.3390/microorganisms9051057

**Published:** 2021-05-13

**Authors:** Abasiofiok Ibekwe, Lisa Durso, Thomas F. Ducey, Adelumola Oladeinde, Charlene R. Jackson, Jonathan G. Frye, Robert Dungan, Tom Moorman, John P. Brooks, Amarachukwu Obayiuwana, Hiren Karathia, Brian Fanelli, Nur Hasan

**Affiliations:** 1 US Salinity Laboratory, Agricultural Research Service, United States Department of Agriculture, Riverside, CA 92507, USA; 2 Agricultural Research Service, United States Department of Agriculture, Lincoln, NE 68583, USA; lisa.durso@usda.gov; 3 Agricultural Research Service, United States Department of Agriculture, Florence, SC 29501, USA; thomas.ducey@usda.gov; 4 Agricultural Research Service, United States Department of Agriculture, Athens, GA 30605, USA; ade.oladeinde@usda.gov (A.O.); Charlene.Jackson@usda.gov (C.R.J.); jonathan.frye@usda.gov (J.G.F.); 5 Agricultural Research Service, United States Department of Agriculture, Kimberly, ID 83341, USA; robert.dungan@usda.gov; 6 Agricultural Research Service, United States Department of Agriculture, Ames, IA 50011, USA; tom.moorman@asda.gov; 7 Agricultural Research Service, United States Department of Agriculture, Mississippi State, MS 39762, USA; john.brooks@usda.gov; 8 Department of Biological Sciences (Microbiology Option), Augustine University Ilara (AUI), Epe 106101, Lagos State, Nigeria; amarachukwu.obayiuwana@augustineuniversity.edu.ng; 9 CosmosID Inc., Rockville, MD 20850, USA; hiren@cosmosid.com (H.K.); brian.fanelli@cosmosid.com (B.F.); hasan@ezbiome.com (N.H.); 10 Center for Bioinformatics and Computational Biology, University of Maryland, College Park, MD 20878, USA

**Keywords:** antimicrobial resistance, extended-spectrum β-lactamase, animal sources, whole-genome sequencing, multi-locus sequence typing

## Abstract

Antimicrobial resistance associated with the spread of plasmid-encoded extended-spectrum β-lactamase (ESBL) genes conferring resistance to third generation cephalosporins is increasing worldwide. However, data on the population of ESBL producing *E. coli* in different animal sources and their antimicrobial characteristics are limited. The purpose of this study was to investigate potential reservoirs of ESBL-encoded genes in *E. coli* isolated from swine, beef, dairy, and poultry collected from different regions of the United States using whole-genome sequencing (WGS). Three hundred isolates were typed into different phylogroups, characterized by BOX AIR-1 PCR and tested for resistance to antimicrobials. Of the 300 isolates, 59.7% were resistant to sulfisoxazole, 49.3% to tetracycline, 32.3% to cephalothin, 22.3% to ampicillin, 20% to streptomycin, 16% to ticarcillin; resistance to the remaining 12 antimicrobials was less than 10%. Phylogroups A and B1 were most prevalent with A (*n* = 92, 30%) and B1 (87 = 29%). A total of nine *E. coli* isolates were confirmed as ESBL producers by double-disk synergy testing and multidrug resistant (MDR) to at least three antimicrobial drug classes. Using WGS, significantly higher numbers of ESBL-*E. coli* were detected in swine and dairy manure than from any other animal sources, suggesting that these may be the primary animal sources for ESBL producing *E. coli*. These isolates carry plasmids, such as IncFIA(B), IncFII, IncX1, IncX4, IncQ1, CollRNAI, Col440I, and acquired ARGs *aph*(6)-Id, *aph*(3″)-Ib, *aad*A5, *aph*(3′)-Ia, *bla*_CTX-M-15_, *bla*_TEM-1B_, *mph*A, *erm*B, *cat*A1, *sul*1, *sul*2, *tet*B, *dfr*A17. One of the *E. coli* isolates from swine with ST 410 was resistant to nine antibiotics and carried more than 28 virulence factors, and this ST has been shown to belong to an international high-risk clone. Our data suggests that ESBL producing *E. coli* are widely distributed in different animal sources, but swine and dairy cattle may be their main reservoir.

## 1. Introduction

Extended-spectrum β-lactamases (ESBLs) are plasmid-encoded enzymes providing resistance to third-generation cephalosporins, which are a class of β-lactam antibiotics that can be used for the treatment of human infections caused by Gram-negative bacteria, especially *Escherichia coli* [1]. Unfortunately, increasing prevalence of infections caused by *E. coli* isolates producing extended-spectrum β-lactamases (ESBLs) have rendered the use of third generation cephalosporins increasingly ineffective against this pathogen [2]. These ESBL-encoding plasmids frequently carry genes encoding resistance to other drug classes, such as fluoroquinolones, aminoglycosides, sulfa derivatives, and trimethoprim [3,4]. ESBL production has been observed mostly in Enterobacteriaceae, particularly *E. coli* and *Klebsiella pneumoniae*, but all other clinically relevant Enterobacteriaceae species are also potential ESBL-producers.

Although in the United States most cephalosporins are restricted for use in humans, ceftiofur, a third-generation cephalosporin, is approved for use in food animals. Marketed under a variety of brand names such as Excede^®^ and Naxcel^®^, it is used therapeutically in chickens, turkeys, dairy cattle, beef cattle, swine, goats, and sheep. Since the use of antibiotics provides selection pressure for the development of resistant microorganisms, farms are therefore an important location for monitoring of antibiotic-resistant bacteria (ARB) and antibiotic resistance genes (ARGs), including genes in commensal bacteria [5]. ARB can be transferred back and forth from animals to humans by direct contact, and can spread to soil, food, and groundwater through the application of manure to agricultural fields [6]. Bacteria from manure can potentially transfer resistance genes horizontally to resident pathogens and commensal microorganisms in soil, even when manure bacteria do not persist in the environment [7].

*E. coli* is frequently identified as carrying β-lactamases enzymes, including ESBLs, resulting in difficulty treating infections, such as urinary tract infections, pneumonia or even sepsis in humans [8]. ESBL-producing *E. coli* have been recognized in veterinary medicine as causative agents of mastitis in dairy cattle since the 2000s [9,10] but few studies exist that have investigated the prevalence of ESBL-producing bacteria in livestock, comparing their existence in sick and/or healthy cattle [11,12]. *E. coli* can become resistant to extended-spectrum cephalosporins through mutations due to the overproduction of AmpC and/or by expression of acquired ESBLs [13]. The genes encoding these acquired enzymes are associated with plasmids with the potential for horizontal dissemination. Plasmid-mediated transfer of drug resistance-encoding genes among bacterial species is one of the most important mechanisms driving the dissemination of multi-drug resistance [14] and the use of third-generation cephalosporin antimicrobial compounds in human and veterinary medicine is considered by some as a risk factor for selection and dissemination of resistant bacterial clones [13,15]. ESBL detection involves two important steps; a screening test with an indicator cephalosporin and a confirmation test which evaluates the synergy between an oxyanion cephalosporin and clavulanic acid [16].

Recent studies have suggested that *E. coli* strains and their associated antibiotic resistance genes can spread from food-producing animals via the food-chain to humans, through exchange of plasmids between multiple animal and environmental reservoirs [17]. Genomic studies note that the highly dynamic genome structure of pathogenic and commensal *E. coli*, built on its strong “clonal frame” predisposes it to constant genetic insertions and deletions [18]. This genomic plasticity is a factor that contributes to its importance as a vector for acquired antibiotic resistance. Additionally, since food animal production encompasses both primary and secondary habitats of *E. coli* (the lower gastrointestinal tract of warm-blooded animal hosts and soil, water, and air) [19], the study of antibiotic resistance in this organism has the potential to elucidate links between food production animals, the environment, and human health. For example, the presence of the IncK2 plasmid in diverse *E. coli* from both human urine isolates and poultry meat production suggested that the IncK2 plasmids originated from a common progenitor, demonstrating the capability of this mobile element to spread to genetically diverse *E. coli* in different reservoirs [20].

In this study, we investigate agricultural *E. coli* as a potential reservoir of antibiotic resistance genes, including AmpC and ESBL-encoding genes, and compared resistance profiles across phylogroups and commodities using traditional and whole genome sequencing (WGS) methods. WGS has been shown to provide superior resolution over traditional typing methods [21,22,23] for the typing of ESBL producing *E. coli*. In this study, we hypothesized that *E. coli* from different animal sources will produce distinctive resistance profiles. Three hundred isolates were typed by PCR into phylogroups, characterized by BOX AIR-1 PCR, and evaluated phenotypically for antibiotic resistance to a panel of 18 drugs. Because of the importance of the ESBL phenotype, additional characterization of ESBL carriage and WGS of ESBL isolates was performed.

## 2. Materials and Methods

### 2.1. Strains in Study and Isolation Method

About 300 *E. coli* isolates from California (CA), North Carolina (NC), Nebraska (NE), North Dakota (ND), Washington (WA), Georgia (GA), Kentucky (KY), Wisconsin (WI), Connecticut (CT), Idaho (ID), Illinois (IL), and South Carolina (SC), were used for this study. The isolates were from beef, dairy, swine, poultry, fish, horse, and lamb manure as well as sediment and surface water ([24], Supplementary Table S1). Supplementary Table S1 provides additional details about commodity sources and locations of samples. Geographic origin, number of isolates, and isolation methods of bacteria from each commodity were as previously described ([24], Supplementary Table S1A). Isolates were confirmed as *E. coli* using API20E strips (bioMérieux, Paris, France), and were genetically confirmed using the *uidA* primer pair [25]. Individual colonies of pure cultures that were isolated were stored at –80 °C for further characterization [26].

### 2.2. Typing of E. coli Using BOX AIR-1 PCR

Genomic DNA fingerprinting of *E. coli* isolates was performed as previously described [27,28,29]. Repetitive Extragenic Palindromic-PCR (REP-PCR) was used to assess the genetic diversity of *E. coli* isolates (Supplementary Table S1B). Rep-PCR fingerprints were obtained by using the primer BOX AIR (5′- primers REP 1R (5′-IIIICGICGICATCIGGC-3′) and REP 2I (5′-ICGICTTATCIGGCCTAC-3′) [30,31]. Following amplification, the PCR amplicons were electrophoresed, and the gel images were obtained using a quality one gel imaging system (Bio-Rad Lab., Hercules, CA, USA). Comparison of restriction enzyme digestion patterns and cluster analysis was performed with the BioNumerics software, version 7.5 (Applied Maths, Austin, TX, USA). Fingerprints were clustered by using the Jaccard coefficient evaluated by the unweighted-pair group method (UPGMA).

### 2.3. Phylogroup Identification

Phylogroups were determined for each *E. coli* isolate using an established multiplex PCR targeting *arp*A (400 bp), *chu*A (288 bp), *yja*A (211 bp), and *tsp*E4.C2 (152 bp) according to the protocol of Clermont et al. [32] for the quadruplex assay. For group E, C, and internal control, the primers *arp*A (301 bp), *trp*A (219 bp) and *trp*A internal control primers *trp*A (489 bp) were used. The method was previously developed to classify *E. coli* into four phylogenetic groups designated A, B1, B2, and D [32], and modified into eight phylogroup structures: seven (A, B1, B2, C, D, E, F) belong to *E. coli* sensu stricto, whereas the eighth is the *Escherichia* cryptic clade I [33].

### 2.4. Susceptibilities of Isolates against 18 Antibiotics

Antimicrobial susceptibility tests (phenotypes) of *E. coli* isolates were assessed using disk diffusion assays following CLSI standards [34] for 18 antimicrobials. The Mueller–Hinton II agar (Difco, Sparks, MD, USA) was used, and cells were harvested from the surface of the medium with a cotton swab after 24 h growth at 37 °C. *E. coli* ATCC 25922 (American Type Culture Collection, Manassas, VA, USA) was included in each assay as a control strain. Antimicrobial agents were tested with BD BBL Sensi-Disc antimicrobial susceptibility test discs (Becton Dickinson & Co., Sparks, MD, USA) with the breakpoints (μg mL^−1^) indicated (Table S2). Positive control (*E. coli* ATCC 25922, *Pseudomonas aeruginosa* ATCC 27853) and negative control *Staphylococcus aureus* ATCC 29213 and *Enterococcus faecalis* ATCC 29212 [33] were included.

### 2.5. Identification of ESBL E. coli

In order to identify isolates for whole genome sequencing, multiplex PCR screens were performed on 300 *E. coli* isolates targeting sequences of genes encoding *bla*_CTX-M_, *bla*_TEM_, *bla*_OXA_ and *bla*_SHV_. Details of primers, annealing temperatures, and amplicon sizes are as previously provided [35]. The multiplex PCR screens were performed using 25 μL mixtures and Ready-To-Go PCR beads master mix (GE Healthcare, Buckinghamshire. UK). Initial screening of ESBL production by 300 isolates was performed on Tryptone Bile X-Glucuronide (TBX) supplemented with 4 mg/L cefotaxime (TBX-CTX). We used TBX because phenotypic detection of ESBLs can be obscured by AmpC-producing bacteria in environmental samples. The isolates were later tested phenotypically for ESBL production by combination disc synergy tests using cefotaxime and ceftazidime with and without clavulanic acid (Becton Dickinson) according to CLSI guidelines [36]. A cefoxitin disc (30 mg, Becton Dickinson) was added to this test, to detect AmpC phenotypes. Unlike ESBLs which are frequently plasmid encoded, AmpC β-lactamases are generally located on the chromosome, and confer resistance to third generation cephalosporins, and oxyimino-monobactams (aztreonam), but not cephamycin or carbapenems. They are classified as Ambler Class C and Bush Jacoby group 1, and although they are not classified as ESBLs, we have included them in our analysis of our agricultural and environmental *E. coli* strain set. All isolates classified as intermediate or resistant using CLSI criteria (≤17 mm) to cefoxitin were suspected to be AmpC producers [36]. Based on the results of the phenotypic test, strains designed as putative ESBL producers were further analyzed by PCR for genes encoding ESBL genotypes: TEM, OXA, SHV, and CTX-M [37,38,39,40,41]. A strain of ESBLs-producing *Klebsiella pneumoniae* (ATCC 700603) was used as positive control for ESBLs gene screening as well as standard strain *Escherichia coli* (ATCC 25922).

### 2.6. Whole Genome Sequencing and Genome Assembly and Analysis

Genomic DNA was extracted with the QIAamp DNA Mini Kit and plasmid with Qiagen Plasmid Mini Kit (Qiagen, Valencia, CA, USA). Samples were quantified using a fluorometer Qubit 3.0 and each sample was normalized in 3–18 µL of nuclease-free water for a final concentration of 0.5 ng µL^−1^ using the Biomek FX liquid handler (Beckman Coulter Life Sciences, Brea, CA, USA). Libraries were then constructed using the modified Nextera XT protocol (Illumina, San Diego, CA, USA) as previously described [42]. PCR products were purified using 1.0× speed beads and eluted in 15 µL of nuclease-free water and quantified by PicoGreen fluorometric assay (100× final dilution). The libraries were pooled by adding an equimolar ratio of each based on the concentration determined by PicoGreen, and loaded onto a high sensitivity (HS) chip run on the Caliper LabChipGX (Perkin Elmer, Waltham, MA, USA) for size estimation, followed by 150 bp paired end sequencing using Illumina HiSeq v3 chemistry (Illumina, San Diego, CA, USA). Sequencing reads were directly analyzed using the CosmosID bioinformatics software package (CosmosID Inc., Rockville, MD, United States) as described previously [43,44,45,46].

Raw sequencing data were trimmed, and de novo assembled using the SPAdes assembler (http://bioinf.spbau.ru/spades accessed on 18 November 2018 [47]) and plasmSPAdes accessed on 18 November 2018 [48] using default parameters to construct each genome. Contigs less than 200 nucleotides were excluded from the analysis. Assembled contigs were submitted to the Center for Genomic Epidemiology’s ResFinder [49] and CARD for the identification of resistance genes carried on plasmids or chromosome [50], and to determine the incompatibility (*inc*) group of the plasmid carrying an ARG of interest. Contigs were also submitted to PlasmidFinder [51] to determine existing plasmid replicon types, and steps previously described [52]. A phylogenetic tree of the sequenced *E. coli* genomes, along with additional reference *E. coli* genomes, was constructed using the parsnp program (Harvest software) [53] which identifies core genomes across isolates and builds a phylogeny using maximum likelihood and core single nucleotide polymorphisms (SNPs). Sequence typing of each genome was performed using MLSTcheck developed by the Sanger Institute, using the pubMLST database (https://pubmlst.org/ accessed on 18 November 2018) as described elsewhere [54]. Draft genomes were submitted to NCBI Short Read Archive under the bio-project #PRJNA492317 (http://www.ncbi.nlm.nih.gov/bioproject/492317, accessed on 18 November 2018). Using Illumina sequencing, there are limitations with a short read assemble in that it is difficult to resolve the entire plasmid into one contig [55,56,57,58]. Consequently, a plasmid is broken down into multiple contigs including the region used for determining plasmid incompatibility group (*inc*RNAi).

Draft assemblies were interrogated against CosmosID acquired antibiotic resistance gene and virulence gene databases using the BLASTN (v.2.7) tool. The best-matching genes were identified using a threshold of >90% identity and >60% alignment coverage of the reference gene. When the *inc*RNAi-*rep* region was absent in a contig carrying AR, then it was not possible to determine the plasmid *inc* group. Protein annotation of contigs were performed using a Prokka [59] and PSI-BLAST search against the National Center for Biotechnology Information (NCBI) database. The genetic context of *bla_TEM_* genes was determined using linear maps of contigs drawn using SnapGene ^®^.

MAFFT v. 1.4.0^ref^ and RAxML v. 4.0 [60] implemented in Geneious Prime^®^ v 2020.0.1 were used for aligning *bla_CMY-2_* plasmid contigs and for reconstructing their maximum likelihood (ML) tree. The GTR + GAMMMA model was used for building the tree implemented. Lastly, to determine the consensus sequence for incA/C2, i.e., incC plasmid present in ARS-isolate-13, we aligned its assembled whole genome against the closest IncC reference genome found on NCBI (Genbank number: CP051316, query cover = 98%; identity = 99.99%) using Geneious Prime^®^ mapper (settings—high sensitivity). Contigs matching the incC reference genome (# = 13) were ordered and annotated with the Rapid Annotation using Subsystem Technology (RAST) [61,62,63]. Virulence genes encoded on incC were determined using VirulenceFinder^ref^. A linear map of IncC was built using the SnapGene ^®^ viewer v. 5. 2.3.

## 3. Results and Discussion

In this study, 300 isolates of *E. coli* were typed by PCR into phylogroups, characterized by BOX AIR-1 PCR (Supplementary Table S1B), and evaluated phenotypically for antibiotic resistance to a panel of 18 antibiotics (Supplementary Figure S1A,B). Only 2% of the dairy cattle *E. coli* isolates (2 isolates out of 98), and 7% of the swine isolates (7 isolates out of 100) in our strain set were positive for our selected ESBL-associated genes when assayed by PCR and displayed the ESBL phenotype in the culture-based double synergy test (Supplementary Figure S2 and Table S2). ESBL phenotypes and/or genotypes were not detected in any of the remaining animal isolates (Supplementary Figure S3). The most frequently detected subtypes were *bla*_CTX-M-1_ and *bla*_CTX-M-9_ (Supplementary Table S3). Nine strains (seven isolated from swine production systems and two from dairy) were identified as ESBL *E. coli* phenotype. Because of the importance of the ESBL phenotype, additional characterization of ESBL carriage and WGS of ESBL isolates was performed.

### 3.1. Whole Genome Sequencing of ESBL Isolates

The genomes of 20 ESBL-producing *E. coli* were sequenced, including nine isolates (*n* = 7 from swine, *n* = 2 from dairy) that were positive for ESBL production by the modified double synergy test. The remaining eleven isolates were PCR positive for *bla*_TEM_, *bla*_CTX-M1_, *bla*_CTX-M9_, *bla*_OXA_ and *bla*_SHV_-like genes, including two isolates from horse and lamb that were not positive for ESBL genes by PCR.

WGS assembly statistics of the draft genomes yielded an average assembled size ranging from 4.7 to 6.6 Mbp and consisting of 81 to 2169 contigs with a mean N50 of 109 kbp. Sequence typing (Supplementary Table S4) revealed all isolates harbored distinct ST types, except isolate-16 (ST 10) and 18 (ST 2). Isolate 16 from poultry belonged to phylogroup D while isolate 18 from a horse belonged to the phylogroup A. These two isolates had different antibiotic resistance phenotypes (none determined in isolate 16) and different antibiotic resistance genes from WGS as shown in isolate 18 (Table 1).

Genes encoding resistance to nine classes of antibiotics were detected by WGS in the 20 *E. coli* isolates sequenced (Table 1). Twelve isolates were resistant to ≥three antibiotics phenotypes, and these were from beef (2), dairy (2), poultry (1), and swine (7). As shown in Table 1, the antibiotic resistance phenotypes on these eight isolates matched very well with the identification of resistance genes based on WGS. Overall, the twelve isolates with MDR phenotypes correlated well with most genotypes. All the isolates from swine carried β-lactam resistance genes. The *bla*_CMY-2_ gene was found in five isolates and was the most common, followed by *bla*_TEM-1_ in five isolates. Only one isolate from swine contained *bla*_CTX-M-15_ and no other CTX-M-type ESBLs were identified. All the isolates that carried any of the β-lactam resistance genes were also MDR. A double synergy test of these isolates confirmed the ESBL phenotype for all the swine isolates used in WGS, and two dairy isolates. Six additional isolates also expressed resistance phenotypes but the corresponding ARGs were not detected by WGS (Table 1).

The presence of 1837 VF genes was detected in the 20 isolates used in this study based on WGS (Supplementary Table S5). The poultry isolates contained higher numbers of VF genes than any other animal source used in this study, while the horse isolates contained the lowest. There were no differences in the number of VF genes detected among the other four animal sources (beef, dairy, swine, lamb) and sediment. The most prevalent VF genes in the isolates were the *flg*, *fli*, *fim*, *che*, and the *csg* genes (Supplementary Table S6). Variants of these genes were present in all the isolates as seen in Supplementary Table S5. One of the genes of interest that was present in most of the isolates was α-hemolysin (*hly*) that included *hly*ABCDE with *hly*E as the most prevalent. Swine and poultry were two of the animal sources that carried *hly*ABCDE genes. The *eae* gene was also identified in all the food animal isolates and sediment, but not in the horse and lamb isolates. The cytotoxic necrotizing factor (CNF) was detected in one isolate from a dairy cow, and this isolate was not ESBL positive. None of the ESBL positive dairy isolates carried the CNF. The *hly* genes were detected in most of the ESBL positive isolates from both dairy and swine.

### 3.2. Plasmids Carrying β-Lactam Resistance

**Figure 1:**
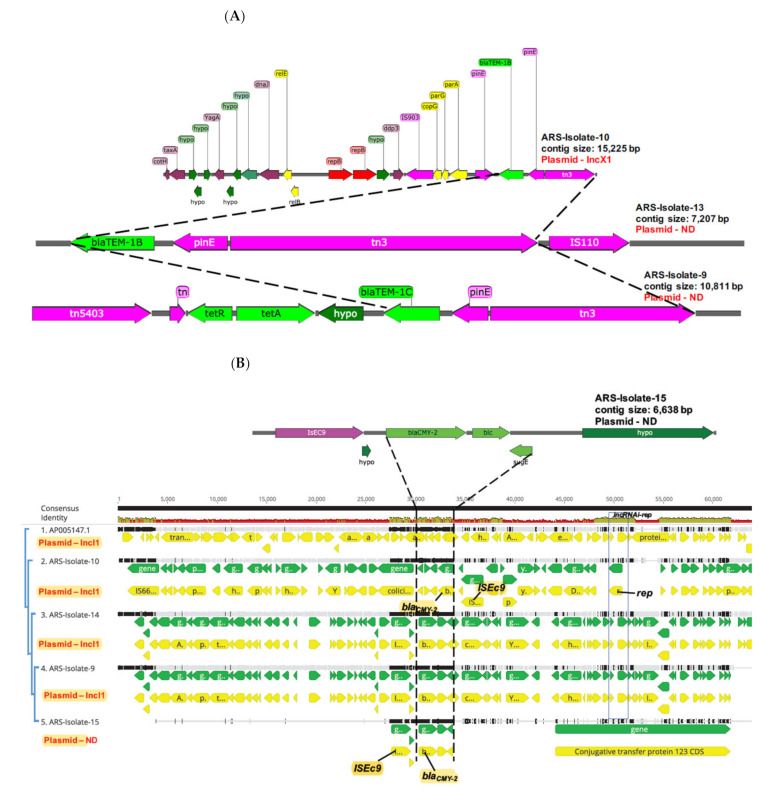

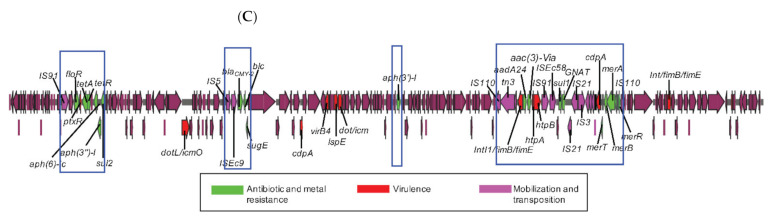
Plasmids carrying β-lactam resistance. (**A**) Plasmid contigs carrying *bla_TEM_* genes in three *E. coli* isolates (10, 13, 9) from this study. Thick dashed lines denote regions that are similar between the three plasmid contigs. (Note: ND, not determined.) (**B**) Alignment of putative IncI1 plasmid contigs carrying *bla_CMY-2_* present in selected *E. coli* isolates from this study. *Bla_CMY-2_* carrying contigs were aligned to a complete IncI1 plasmid (R64; Genbank number—AP005147). The tree on the left was built using the GTR model of nucleotide substitution and the GAMMA model of rate heterogeneity. Horizontal dashed lines highlight the genetic context of *bla_CMY-_*_2_ in these plasmids, whereas the blue rectangular box shows the region encoding *inc*RNAi and the replication initiation protein (*rep*) in 4 of 5 plasmid contigs. (Note: mean pairwise DNA identity of the contigs are shown in green-brown (at least 30 % and under 100% identity) and red (below 30 % identity) bars; ND, not determined.) (**C**) Linear map of a multidrug resistant incA/C2 (incC) plasmid (~166,736 bp) present in one *E. coli* isolate from this study. Contigs (*n* = 13) matching the closest IncC reference genome found on NCBI (Genbank number: CP051316; >99% pairwise DNA identity) was concatenated and ordered with the reference genome. Genes encoding ARG, virulence and mobile genetic elements are colored green, red and magenta, respectively. The blue rectangular box highlights putative mobile regions encoding antimicrobial resistance genes.

In this study, we aligned the contigs present in isolates carrying the *bla*_TEM_ gene. For instance, isolate 10 carries the *bla_TEM-1B_* gene between two transposases/recombinases (*pinE* and *tn3*). For this isolate, the *bla_TEM-1B_* gene is present on the same contig with *inc*RNAi, therefore, we are sure this is an IncX1 plasmid (Figure 1A). For isolates 9 and 13, unfortunately, the contig that carries the *bla*_TEM_ gene does not have the *inc*RNAi region, thus longer reads will be required to confirm the *inc* group for these plasmids. Nevertheless, the region carrying *bla*_TEM_ for the three plasmids share significant DNA homology and arrangement (*bla*_TEM_—*pinE-tn3*).

To determine the genetic context of the *bla*_CMY-2_ gene, we aligned putative IncI1 contigs carrying the *bla*_CMY-2_ gene to a complete circular R64 IncI1 plasmid (Genbank number: AP005147). The *bla*_CMY-2_-*blc*-*sugE* genetic backbone (dashed horizontal lines) was conserved in all plasmids and all isolates except ARS-Isolate-15 carried the *bla*_CMY-2_ gene on the same contig that harbored the *inc*RNAi (blue rectangular box). This result confirms that 3 of the 4 isolates carry the *bla*_CMY-2_ on a IncI1 plasmid but we cannot ascertain their complete size (Figure 1B). Furthermore, we identified 13 contigs totaling ~166 kbp that matched the multidrug resistant IncA/C2 (IncC—Genbank: CP051316) plasmid (largest contig—75, 063 bp; smallest contig—154 bp) in isolate 13 that carries *tetA*, *tetR*, *aph(3″)-I* (*strA)*, *aph(6)-Ic* (*strB)*, *floR, sul2, bla*_CMY-2_-*blc*-*sugE, aadA24, aac(3)-Via* and the *sul1* gene (Figure 1C). In addition to ARG’s, the incC plasmid harbors virulence and metal genes (mer operon). In a study with poultry flocks, Zurfluh et al. [64] showed that some genetically similar IncI1 plasmids were found in ESBL-producing *E. coli* of different MLST types isolated at the different levels in the broiler production system. Their data, based on comparative sequence analysis, highlighted the successful spread of bla_ESBL_ harboring plasmids of different Inc types among isolates of human and food-producing animal origin and provide further evidence for potential dissemination routes [37,65]. Furthermore, ESBL-encoding *Escherichia coli* cultured from pigs and their plasmids characterized, and their data showed all seven isolates carried one or more high-molecular-weight plasmids and demonstrated the ability to transfer their cefotaxime resistance phenotype at high frequencies. Five transmissible plasmid replicon types were detected, including IncK/B (n¼3), IncI1 (n¼2), IncFIA (n¼1), IncFIB (n¼1), and IncN (n¼1). ESBL-encoding genes, including *bla*_CTX-M-14_, *bla*_CTX-M-15_ and *bla*_TEM-20_, were identified [37]. In our study, no *bla*_ESBL_ producing *E. coli* was identify in poultry and lamb. However, Wang et al. [65] did some analysis of accessory genes in 14 conjugative plasmids from nine unrelated human, poultry and lamb *E. coli* isolates and found that these isolates can transfer their *bla*_ESBL_ genes to other bacterial strains. They reported that insertion sequences and transposons were the likely tool for the dissemination of the *bla*_CTX−M−1_ and *bla*_TEM_ gene between different environments.

### 3.3. Phylogenetic Analysis

**Figure 2:**
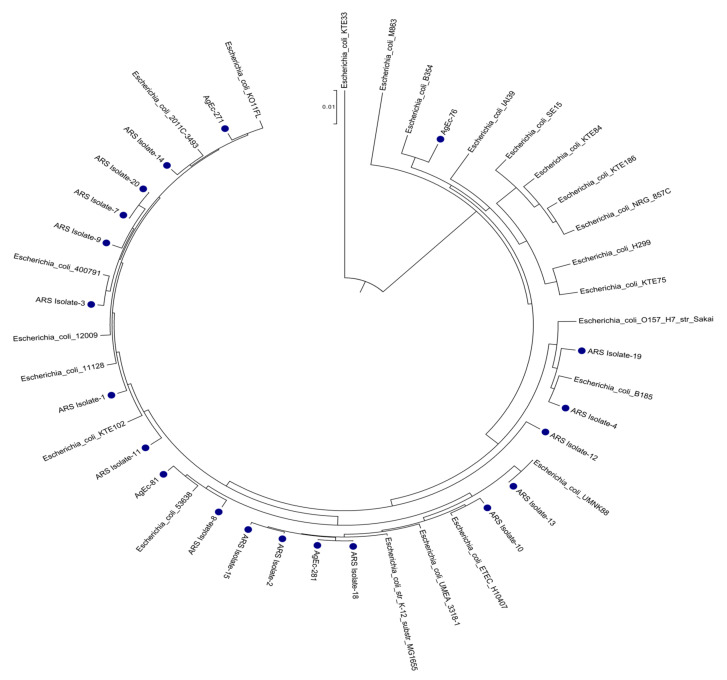
Core genome SNP-based phylogenetic analyses of *E. coli* including ESBL-positive strains sequenced for this study. The strains sequenced in this study are indicated in colored bullets.

The genome sequences of the 20 newly sequenced *E. coli* isolates were compared with an additional 24 publicly available reference *E. coli* genomes to determine their evolutionary relatedness, using core genome SNP-based phylogenetic analyses (Figure 2). The derived *E. coli* tree demonstrated fully resolved bifurcating patterns with varying levels of diversity and placed these newly sequenced genomes into paraphyletic clades, suggesting distinct evolutionary lineages of these *E. coli* genomes. De novo assembled sequences were identified as *E. coli* through a CosmosID metagenomic analysis via app.cosmosid.com. A selection of 23 reference *E. coli* genomes to represent a range of the species, primarily completed genomes, were chosen through the NCBI assembled genome database (Table S7) with each genome’s name within the SNP tree, along with the GenBank accession, refseq accession, and ST type (both the Achtman Schema #1 and Pasteur Schema #2). Most of the isolates were placed into district clades of *E. coli* genomes pathogenic to human and animals (i.e., ETEC, EPEC, EHEC, EIEC, etc.). For example, isolate-19 and 4 on the SNP tree formed a monophyletic clade with *E. coli* O157:H7 str. Sakai, isolate-13 branched with a pig-pathogenic *E. coli* UMNK88, isolate-8 and AgEc-81 were clustered with enteroinvasive *E. coli* 53638, isolate-1 branched with enterohemorrhagic *E. coli* 11128, isolate-3, 7, 9 and 20 branched with enteropathogenic *E. coli* 400791, whereas isolate-14 formed a monophyletic clade with *E. coli* O104:H4 strain 2011C-3493.

The transmission of ESBL genes in agricultural and environmental matrices has significant implications for our understanding of the dynamics of the spread of ESBL genes and for evaluating control measures. Since ESBL *E. coli* are global health threats, it is critical to better understand the ecology of these organisms [66], and information on non-clinical isolates provides important context for understanding the relationships between genotype, phenotype, and the potential for deciphering the evolutionary mechanisms that contribute to transfer of these strains into human pathogens [18]. One component of this is the fate and transport of *E. coli* expressing phenotypic and/or genotypic ESBL resistance from food animals to the environment. To reduce the spread of ESBL *E. coli* and ESBL-encoding genes, we must consider the possible sources and understand the pathway and mechanisms by which resistance is disseminated. This can be attributed to the transfer of the bacterial mobile antibiotic resistance gene (ARGs) across different environmental niches [67]. However, the transfer network of the mobile resistome and the forces driving mobile ARG transfer are unknown. In this study, we considered an important vector of ESBL transfer across ecosystems and the potential role of environmental *E. coli* in ESBL dissemination.

The *bla*_CMY-2_ gene, which is the most common plasmid-mediated *ampC* β-lactamase gene worldwide [17], was the only acquired *ampC* β-lactamase gene detected in this study, and this came from one isolate (isolate #6) from dairy and four isolates from swine (isolates #9, 10, 13, and 15). IS*Ecp*1 insertion sequence upstream of ESBL/pAmpC genes are associated with transposition and chromosomal integration of typically plasmid-encoded genes in *E. coli*, *K. pneumoniae*, and *Shigella flexneri*, among others, from animals or humans [37]. The *bla_CTX_* gene was one of the ESBL gene detected in the isolates from this study and these are also common in clinical *E. coli* isolates collected in Japan, and elsewhere [68].

Therefore, the presence of these genes in environmental isolates is a concern for public health officials trying to understand the fate and transport of AR bacteria from commensal bacteria in the environment to pathogens that can infect humans. ST410 has been identified as a “high-risk” clone which should be monitored closely [56] and this is like another high-risk clone (ST 131) that is globally distributed [69]. *E. coli* ST410 has been reported world-wide as an extraintestinal pathogen associated with resistance to fluoroquinolones, third generation cephalosporins, and carbapenems [56], and was detected in a swine isolate from this study.

### 3.4. Relative Abundances of ARGs and Virulence Factor (VFs) in ESBL E. coli

The relatedness of ESBL-producing *E. coli* from different animal sources and the environment was assessed using WGS. ESBL enzymes have been classified into three major subtypes: TEM, SHV and CTX-M β-lactamases. In animals, the most common genes associated with ESBL resistance are *bla*_CTX-M-1_, *bla*_CTX-M-2_, *bla*_CTX-M-14_, *bla_CTX-M-15_, bla*_TEM-52_ and *bla*_SHV-12_ [70]. The most frequently detected subtypes were *bla*_CTX-M-1_ and *bla*_CTX-M-9_ (Supplementary Table S3). Our data confirmed that ESBL producing *E. coli* is most dominant in swine then followed by cattle, and fewer in poultry. Phylogenetic group A and clinical isolates from phylogroup A and B have been associated with urinary tract infections [71,72,73,74,75,76,77,78], and many studies have determined antibiotic resistance in animal production environment associated with ESBL producing *E. coli* [79,80,81,82,83,84,85]. It should also be noted that non-agricultural environments may produce the same or higher ESBL producing *E. coli* or AR bacteria [86,87,88,89,90,91]. In addition, the transport of pathogens carrying MDR associated with integrons or mobile DNA elements such as plasmids and transposons from animal feces to the environment has been well documented [92,93,94].In a recent review by Ramos et al. [55], these authors examined the global distribution of genes responsible for resistance to extended-spectrum cephalosporins, and confirmed that the CTX-M-1 group (CTX-M-1 and -15) is predominant in European countries, CTX-M-9 and -14 were frequently identify in Spain, Portugal, and the United Kingdom, and the CTX-M-1 group are the most frequent observed in the United States and North Africa. During our study the most common *ESBL* genes were *bla*_CTX-M-1_ and *bla*_CTX-M-9_. Two isolates from dairy carried CTX-M-9 while one isolate carried CTX-M-1. In addition, two isolates from swine carried CTX-M-1, while five isolates carried CTX-M-9. During this study, we did not conduct a conjugation assay to confirm that the cefotaxime resistance marker was successfully transferred to a susceptible *E. coli* as was previously done by Wang et al. [37]. These authors were able to confirm the *bla*_ESBL_ genes transferred by conjugation as *bla*_CTX-M-14_, *bla*_CTX-M-15_, and *bla*_TEM-20_. Others have used the two-step approach, as we did in this study, to identify and confirm ESBL producing *E. coli* [11,17,38,39]. Other studies have also found low numbers of ESBL producing *E. coli* in farm animals. For instance, in a study conducted in Japan, the prevalence of ESBL among cattle was 1.5% [40] and 8.4% in Swiss cattle [41]. However, in a review presented by Ramos et al. [55], these authors showed very high percentages or ESBL producing *E. coli* in pigs, cattle, and poultry ranging from 4% to 90%. Therefore, the prevalence of ESBL *E. coli* may depend on the size of the operation, animal type, and other factors.

In conclusion, we investigated agricultural *E. coli* as a potential reservoir of antibiotic resistance genes, including ESBL-encoding genes, and compared resistance profiles across phylogroups and commodities using traditional and whole genome sequencing (WGS) methods. We detected most of the ESBL producing *E. coli* were from swine and dairy, indicating the importance of these commodities in disseminating ESBL producing *E. coli* in the agricultural environment. We also determined that all of the ESBL positive phenotypes were MDR. One isolate from swine was from ST410 with properties of an international high-risk clone that was resistant to more than 12 antibiotics and carrying 12 ARGs. The transfer of this strain from swine manure to the environment is of great concern, and therefore, any strategy that reduces the transfer of ARGs from animal manure to the environment should be the first option in any mitigation program. Furthermore, due to the particular ecology of *E. coli* in animals and the environment, it has a unique potential that may help uncover links between agricultural production and human health.

## Figures and Tables

**Table 1:** ESBL and other β-lactamase encoding plasmids and ARGs detected in *E. coli* strains sequenced in this study.

Isolate ID	Source	Phylogroup	ST-Type	Plasmids Identified *	Resistance Phenotype ^a^	Acquired ARG ^b,^*	Point Mutation
Isolate_15-ESBL+	swine	C	6913	IncFII, IncI1, ColRNAI	AMC, AM, AZM, S, TE	aac(3)-IV, aadA1, aadA2, *bla*_CMY-28*_, cml, sul3, tetA, dfrA15	None
Isolate_13-ESBL+	swine	D	100	IncFIB, IncFII, IncFIC, IncI1, **IncA/C2**, Col440I	AM, C, GM, K, NA, S, TE, TIC	aph(3′)-Ia, aph(3′’)-Ib, aph(6)-Id, aadA24, aac(3)-Via, *bla*_CMY-28*_, *bla*_TEM-1B_, floR, sul1, sul2, tetA, *str*A	parC p.S80I, gyrA p.S83L
Isolate_12-ESBL+	swine	C	1771	IncFIB, IncX1, IncX4, IncI1, p0111, ColRNAI, Col (MG828)	AM, S, TE	aph(6)-Id, aph(3′’)-Ib, *str*A	None
Isolate_01	beef	B1	327	IncFIB, IncFII, IncX1, IncY, ColRNAI	C, S, G, TE	aph(6)-Id, aph(3′’)-Ib, floR, sul2, tetA, *str*A	None
Isolate_03	beef	A	1101	IncFIB, IncFII	S, G, TE	aph(6)-Id, aph(3′’)-Ib, sul2, tetB, *str*B	None
Isolate_11-ESBL+	swine	C	410	IncFIA(B), IncFII, IncX1, IncX4, IncQ1, CollRNAI, Col440I	AM, AMZ, CRO, CF, C, CIP, K, NA, S, G, TE, TIC	aph(6)-Id, aph(3′’)-Ib, aadA5, aph(3′)-Ia, *bla*_CTX-M-15_, *bla*_TEM-1B_, mphA, ermB, catA1, sul1, sul2, tetB, dfrA17	parE p.S458A, parC p.S80I, gyrA p.S83L, gyrA p.D87N
Isolate_10-ESBL+	swine	D	48	IncX4, **IncX1, IncI1**	AM, AZM, CRO, CF, TE, TIC	aph(6)-Id, aph(3′’)-Ib, ermB, lnuG, tetB, ***bla*_TEM-1B_, *bla*_CMY_**_-2*8,_	None
Isolate_09-ESBL+	swine	B1	711	IncFIA(B), IncX1, **IncI1**, IncI2, ColRNAI, Col(MG828), Col156	AM, CRO, CF, TE, TIC	*Bla*_CMY-28*_, *bla*_TEM-1C_, tetA	None
Isolate_14-ESBL+	swine	B1	101	IncX4, **IncI1**, IncHI2A	AMC, AM, AZM, FOX, TE	*Bla*_CMY-28*_, ermB, tetB	None
Isolate_08	dairy	B1	710	IncFIA(B)	CF, G	None	None
Isolate_02	beef	B1	43	IncFIA(B), IncFII	G	None	None
Isolate_04	dairy	A	1300	IncFIA(B), IncFII, IncX1, ColRNAI	CF	None	None
Isolate_18	horse	A	10	IncFIB, IncFII	S, G	None	None
Isolate_20	sediment	B1	154	IncFIB, IncFII	AZM, G	None	None
AgEc_17	poultry	E	155	IncFIB, IncI1, ColRNAI	AM, AMZ, S, G	*str*B, *mef*(B)	None
Isolate_16	poultry	D	10	ND	ND	ND	ND
Isolate_5-ESBL+	dairy	A	2	IncFIB, IncFII	CF, G	*str*B	parC p.S57T
Isolate_6-ESBL+	dairy	D	685	IncI1, IncY	CF, G, TIC	None	None
Isolate_07	dairy	D	154	IncFIB, IncFII, IncX1,IncI1, ColRNAI	CF, G, TIC	tetA	None
Isolate_19	lamb	E	6060	IncFII, IncX1, IncI1, ColRNAI	TE	tetC	None

^a^ Amikacin (AN), Amoxicillin and Clavulanic acid (AMC), Ampicillin (AM), Azithromycin (AZM), Cefoxitin (FOX), Ceftriaxone (CRO), Cephalothin (CF), Chloramphenicol (C), Ciprofloxacin (CIP), Gentamicin (GM), Imipenem (IPM), Kanamycin (K), Nalidixic acid (NA), Streptomycin (S), Sulfamethoxazole with Trimethoprim (SXT), Sulfisoxazole (G), Tetracycline (TE), Ticarallin (TIC). ^b^ aminoglycoside (*str, aad,*), Sulfonamide (*sul)*, tetracycline (*tet*), phenicol (*flo*), beta-lactamase (*bla*), macrolide (*erm, mef*), Phenicol (*cat, cml*), trimethoprim (*dfr*), macrolide (*mph*). A double synergy test of these isolates confirmed the ESBL phenotype for all the swine. 378 isolates used in WGS, and two dairy isolates. * Boldness denotes the plasmid incompatibility group carrying ***bla_TEM-1B_*** and/or ***bla_CMY-2_*** gene as shown in Figure 1A. ** CMY-2 codes for a ß-lactamase and is included here, but it is not considered as ESBL.

## Data Availability

The original contributions presented in the study are included in the article/Supplementary Materials, further inquiry can be directed to the corresponding author. Draft genomes were submitted to NCBI Short Read Archive under bio-project #PRJNA492317. http://www.ncbi.nlm.nih.gov/bioproject/492317 accessed on 18 November 2018.

## Supplementary Materials

The Supplementary Materials are available online at https://www.mdpi.com/article/10.3390/microorganisms9051057/s1. Table S1. Animal sources and states where isolates were collected from: The 12 states are CA, CT, NC, ND, WI, ID, NE, WA, GA, KY, SC, IL; Table S1B. Diversity and the distributions of 181 unique genotypes and their detection frequencies from the different animal sources; Table S2: Antimicrobial susceptibility test with their breakpoints (μg mL^−1^); Table S3. Identification of ESBL-phenotype +ve strains by double synergy test; Table S4 A: Sequence typing of E. coli genomes using two established MLST schema: Schema-1 and 2; Table S5: vireluence factor genes and percent identity; Table S6. Distribution of virulence factor genes from different animal sources; Table S7. Twenty three reference E. coli genomes were chosen to represent a range of the species from the NCBI assembled genome database. Below with each genome’s name within the SNP tree, along with the GenBank accession, refseq accession, and ST type (both the Achtman Schema #1 and Pasteur Schema #2); Figure S1. (A) Phylo-group by quadruplex PCR assay. The most prevalent phylo-groups were A and B1;(B) Phylo-group by quadruplex PCR assay. The most prevalent phylo-groups were A and B1, then followed by D and E. Within each animal source, 14.28, 19.00, 42.8 and 8.8% for phylo-group A, were from beef, dairy, swine, and poultry, respectively. Also, 19.7, 20.9, 29.1, and 19.8% for phylo-group B1 were from beef, dairy, swine, and poultry, respectively. The rest of the animal sources and environmental samples showed lower distributions of phylo-groups, although these samples had fewer numbers of isolates. The 12 states that isolates were collected are California (CA), Connecticut (CT), North Carolina (NC), North Dakota (ND), Wisconsin (WI), Idaho (ID), Nabraska (NE), Washington (WA), Giorgia (GA), Kentucky (KY), South Carolina (SC), Illinois (IL); Figure S2. Percent susceptibility of 300 E. coli isolates against18 antibiotics; Figure S3. Detection of ESBL E. coli phenotype by double synergy test. All 300 E. coli isolates were screened for ESBL production on TBX media supplemented with 4 mg/L cefotaxime (TBX-CTX) for detection of ESBL E. coli. A total of nine isolates were positive for ESBL production, and eleven additional isolates from other animal and environmental sources were included for whole genome sequencing. Data marked in color are the ESBL positive isolates (seven from swine and two from dairy). In most instances, the genes coding for the observed phenotypes were identified using WGS. Isolates 12 is from swine and that makes 7 isolates.
