# Supplementary material for: Diversity of Plasmids and Genes Encoding Resistance to Extended-Spectrum β-Lactamase in Escherichia coli from Different Animal Sources
Source: Microorganisms. 2021 May 13;9(5):1057. doi: 10.3390/microorganisms9051057 (PMC8153348; doi:10.3390/microorganisms9051057)
Supplement: Supplementary file 1 [file microorganisms-09-01057-s001.zip › Micro-Table S1-S4-supplimentary data.docx]

**SUPPLIMENTARY DATA**

**Diversity of Plasmids and Genes Encoding Resistance to Extended-Spectrum β-lactamase *Escherichia coli* from different Animal Sources**

A. M. Ibekwe^1^*, L. Durso^2^, T. Ducey^3^, A. Oladeinde^4^, K. Cook^4^, C. R. Jackson^4^, J. G. Frye^4^, R. Dungan^5^, T. Moorman^6^, J. Brooks^7^, H. Karathia^8^, B. Fanelli^8^, N. A. Hasan^8,9^

^1^USDA-ARS, US Salinity Laboratory, Riverside CA USA, ^2^USDA-ARS, Lincoln, NE, ^3^USDA-ARS Florence SC USA, ^4^USDA-ARS Athens, GA, ^5^USDA-ARS Kimberly ID USA, ^6^USDA-ARS Ames Iowa USA, ^7^USDA-ARS Mississippi State, MS USA, ^8^CosmosID Inc., MD, USA, ^9^Center for Bioinformatics and Computational Biology, University of Maryland, College Park, MD, USA.

S.1. Typing of E. coli using REP-PCR

*E. coli* isolates with fingerprint pattern similarity scores above 90% were considered clonal populations and were subtyped using BOX A-IR DNA fingerprinting with Jaccard similarity coefficients and UPGMA (Fig. S1B). All isolates (n =300) were grouped into 181 unique genotypes with Shannon diversity index (H') of 4.88 (Table S1B). The distributions of 181 unique genotypes and their detection frequencies from the different animal sources are presented in Table S1B. The isolates used for this study were from beef (n = 38), dairy (n =98), swine (n = 100) and poultry (n=37). This resulted in 33, 68, 68, and 25 unique genotypes, and with Shannon diversity indices of 3.44, 4.09, 4.05, and 3.12 respectively, from beef, dairy, swine, and poultry. The number of unique genotypes may represent the typical clonal population from the isolates analyzed within each animal source. The rest of the sources had only a small number of isolates as shown in Table 1. The data showed how diverse the isolates were from the various sources based on the Shannon diversity index. Since these isolates were from different parts of the country, it is not surprising to see high diversity values and unique genotypes from each animal source.

Table S1. Animal sources and states where isolates were collected from: The 12 states are CA, CT, NC, ND, WI, ID, NE, WA, GA, KY, SC, IL

| Beef (*n* = 38) | *n*† | |
| --- | --- | --- |
| California | 21 | (55.3) |
| Connecticut | 3 | (7.9) |
| North Carolina | 6 | (15.8) |
| North Dakota | 1 | (2.6) |
| Wisconsin | 7 | (18.4) |
| Dairy (*n* = 98) |  |  |
| California | 29 | (29.6) |
| Connecticut | 5 | (5.1) |
| Idaho | 42 | (42.9) |
| North Carolina | 4 | (4.1) |
| Nebraska | 3 | (3.1) |
| Washington | 3 | (3.1) |
| Wisconsin | 12 | (12.2) |
| Environment (*n* = 16) |  |  |
| Idaho | 6 | (37.5) |
| North Carolina | 10 | (62.5) |
| Fish (*n* = 4) |  |  |
| Idaho | 4 | (100.0) |
| Horse (*n* = 5) |  |  |
| Idaho | 5 | (100.0) |
| Lamb (*n* = 2) |  |  |
| Idaho | 2 | (100.0) |
| Poultry (*n* = 37) |  |  |
| Connecticut | 3 | (8.1) |
| Georgia | 11 | (29.7) |
| Kentucky | 10 | (27.0) |
| North Carolina | 2 | (5.4) |
| Nebraska | 3 | (8.1) |
| South Carolina | 8 | (21.6) |
| Swine (*n* = 100) |  |  |
| California | 12 | (12.0) |
| Connecticut | 4 | (4.0) |
| Idaho | 6 | (6.0) |
| Illinois | 4 | (4.0) |
| North Carolina | 70 | (70.0) |
| Wisconsin | 4 | (4.0) |

**†** Percentage of isolates from each geographic location with the animal source.

**Table S1B.** Diversity and the distributions of 181 unique genotypes and their detection frequencies from the different animal sources.

| **Commodity** | **No. of Isolates** | **No. of Unique Genotypes** | ***H'* Index** |
| --- | --- | --- | --- |
| Dairy | 98 | 68 | 4.09 |
| Swine | 100 | 68 | 4.05 |
| Poultry | 37 | 25 | 3.12 |
| Beef | 38 | 33 | 3.34 |
| Fish | 4 | 1 | 0 |
| Horse | 5 | 4 | 1.33 |
| Lamb | 2 | 2 | 0.69 |
| Sediment | 8 | 5 | 1.60 |
| River | 8 | 7 | 1.90 |
| Total* | 300 | 181 | 4.87 |

*Total number of isolates used to calculate diversity. .

Table S2: Antimicrobial susceptibility test with their breakpoints (μg mL^−1^).

| Antibiotics | Disk content (μg)* | Zone diameter interpretative standard -control (mm)+ | R (mm) | I (mm) | S (mm) |
| --- | --- | --- | --- | --- | --- |
| Amikacin | 30 | 19-26 | ≤14 | 15-18 | ≥17 |
| Amoxicillin/clavulanic acid | 20/10 | 18-24 | ≤13 | 14-17 | ≥18 |
| Ampicillin | 10 | 15-22 | ≤13 | 14-16 | ≥17 |
| Azithromycin | - | - | - | - | - |
| Cefoxitin | 30 | 28-34 | ≤14 | 15-17 | ≥18 |
| Ceftriaxone | 30 | 29-35 | ≤13 | 14-20 | ≥21 |
| Cephalothin | 30 | 15-21 | ≤14 | 15-17 | ≥18 |
| Chloramphenicol | 30 | 21-27 | ≤12 | 13-17 | ≥18 |
| Ciprofloxacin | 5 | 29-37 | ≤15 | 16-20 | ≥21 |
| Gentamicin | 120/10 | 19-26 | ≤12 | 13-14 | ≥15 |
| Imipenem | 10 | 26-32 | ≤13 | 14-15 | ≥16 |
| Kanamycin | 30 | 17-25 | ≤13 | 14-17 | ≥18 |
| Nalidixic acid | 30 | 22-28 | ≤13 | 14-18 | ≥19 |
| Streptomycin | 10 | 12-20 | ≤11 | 12-14 | ≥15 |
| Trimethoprim/sulfamethoxazole | 23.75/1.25 | 23-29 | ≤10 | 11-15 | ≥16 |
| Sulfisoxazole | 250 | 15-23 | ≤12 | 13-16 | ≥17 |
| Tetracycline | 30 | 18-25 | ≤14 | 15-18 | ≥19 |
| Ticarcillin | 75 | 24-30 | ≤14 | 15-19 | ≥20 |

- *Concentration of antimicrobial agents, + Zone diameter interpretation for control: R= resistant, I = intermediate, S = susceptible. These were the ranges used in the study to measure the three parameters.

**Table S3.** Identification of ESBL-phenotype +ve strains by double synergy test.

| **STATES*** | **Source** | **ESBL-PCR+** | **ESBL +ve strains** | ***bla*_TEM_** | ***bla*_CTX_-M1** | ***bla*_CTX_-M9** | ***bla*_OXA_** | ***bla*_SHV_** |
| --- | --- | --- | --- | --- | --- | --- | --- | --- |
| CA | Beef | 17 (1) | - | + | - | + | - | - |
| CA | Beef | 30 (2) | - | - | - | + | - | + |
| CA | Beef | 37 (3) | - | - | - | + | - | - |
| WI | Dairy | 61 (4) | - | - | + | + | - | - |
| CA | Dairy | 76(5) | + | - | + | + | - | - |
| CA | Dairy | 81(6) | + | + | - | + | - | - |
| ID | Dairy | 95(7) | - | - | - | + | - | - |
| ID | Dairy | 96(8) | - | - | - | + | - | - |
| NC | Swine | 198(9) | + | + | - | - | - | - |
| NC | Swine | 200(10) | + | + | - | - | - | - |
| NC | Swine | 210(11) | + | + | - | + | - | - |
| NC | Swine | 214(12) | + | - | - | + | - | - |
| NC | Swine | 217(13) | + | + | + | + | - | - |
| NC | Swine | 225(14) | + | - | - | + | - | - |
| NC | Swine | 226(15) | + | - | + | + | - | + |
| . SC | Poultry | 267(16) | - | + | - | + | - | - |
| SC | Poultry | 271(17) | - | + | + | + | - | - |
| ID | horse | 281(18) | - | - | - | + | - | - |
| ID | lamb | 283(19) | - | - | + | + | - | + |
| ID | sediment | 287(20) | - | - | - | + | - | - |

*CA = California, WI = Wisconsin, ID = Idaho, NC = North Carolina, SC = South Carolina

+ ESBL-PCR: # 17 through 287 are the tracking number from the 300 isolates and #(1) though (20) are the final isolate numbers used for WGS.

Table S4 A: Sequence typing of *E. coli* genomes using two established MLST schema: Schema-1 and 2**.**

| **MLST Schema-1** |  |  |  |  |  |  |  |  |  |  |
| --- | --- | --- | --- | --- | --- | --- | --- | --- | --- | --- |
|  |  |  |  |  |  |  |  |  |  |  |
| Isolate | ST | New **ST** | adk | fumC | gyrB | icd | mdh | purA | recA |  |
| Isolate_01 | 327 |  | 6 | 4 | 4 | 85 | 43 | 12 | 7 |  |
| Isolate_02 | 43 |  | 24 | 11 | 4 | 8 | 8 | 8 | 2 |  |
| Isolate_03 | 1101 |  | 9 | 8 | 5 | 1 | 9 | 8 | 7 |  |
| Isolate_04 | 1300 |  | 12 | 136 | 199 | 30 | 24 | 2 | 17 |  |
| Isolate_07 | 154 |  | 6 | 6 | 5 | 10 | 9 | 8 | 6 |  |
| Isolate_08 | 710 |  | 6 | 153 | 4 | 91 | 7 | 8 | 6 |  |
| Isolate_09 | 711 |  | 9 | 6 | 15 | 131 | 24 | 7 | 7 |  |
| Isolate_10 | 48 |  | 6 | 11 | 4 | 8 | 8 | 8 | 2 |  |
| Isolate_11 | 410 |  | 6 | 4 | 12 | 1 | 20 | 18 | 7 |  |
| Isolate_12 | 1771 |  | 224 | 4 | 54 | 247 | 11 | 1 | 7 |  |
| Isolate_13 | 100 |  | 10 | 27 | 5 | 10 | 12 | 9 | 2 |  |
| Isolate_14 | 101 |  | 43 | 41 | 15 | 18 | 11 | 7 | 6 |  |
| Isolate_15 | 6913~ | Unknown | 10 | 932 | 4 | 8 | 8~ | 8 | 2 |  |
| Isolate_16 | 10~ | Novel ST | 10 | 11 | 4 | 8 | 8 | 23 | 2 |  |
| Isolate_19 | 6060 |  | 179 | 789 | 225 | 286 | 15 | 2 | 2 |  |
| Isolate_20 | 154~ | Unknown | 6 | 6~ | 5 | 10 | 9 | 8 | 6 |  |
| AgEc_271 | 155 |  | 6 | 4 | 14 | 16 | 24 | 8 | 14 |  |
| AgEc_18 | 10~ | Novel ST | 10 | 11 | 4 | 8 | 8 | 23 | 2 |  |
| AgEc_76 | 2~ | Unknown | 5 | 3 | 2 | 6 | 5 | 5 | 4~ |  |
| AgEc_81 | 685 |  | 8 | 11 | 4 | 8 | 8 | 8 | 2 |  |

|  | |  | | |  | | |  | | |  | | |  | | |  | | |  | | |  | | |  | | |  | | |  |
| --- | --- | --- | --- | --- | --- | --- | --- | --- | --- | --- | --- | --- | --- | --- | --- | --- | --- | --- | --- | --- | --- | --- | --- | --- | --- | --- | --- | --- | --- | --- | --- | --- |
| MLST Schema 2 | |  | | |  | | |  | | |  | | |  | | |  | | |  | | |  | | |  | | |  | | |  |
| Isolate | | ST | | | New **ST** | | | dinB | | | icdA | | | pabB | | | polB | | | putP | | | trpA | | | trpB | | | uidA | | |  |
| Isolate_01 | | 545 | | |  | | | 21 | | | 34 | | | 3 | | | 23 | | | 6 | | | 1 | | | 15 | | | 2 | | |  |
| Isolate_02 | | 2 | | |  | | | 8 | | | 2 | | | 7 | | | 3 | | | 7 | | | 1 | | | 4 | | | 2 | | |  |
| Isolate_03 | | 227~ | | | Unknown | | | 5 | | | 3 | | | 4~ | | | 10 | | | 26 | | | 108 | | | 4 | | | 110 | | |  |
| Isolate_04 | | 113~ | | | Unknown | | | 22~ | | | 42 | | | 128~ | | | 39 | | | 38 | | | 55~ | | | 127~ | | | 38~ | | |  |
| Isolate_07 | | 338 | | |  | | | 24 | | | 3 | | | 3 | | | 26 | | | 16 | | | 108 | | | 4 | | | 2 | | |  |
| Isolate_08 | | 499~ | | | Unknown | | | 103 | | | 196 | | | 7 | | | 3 | | | 16 | | | 1 | | | 4 | | | 2~ | | |  |
| Isolate_09 | | 86~ | | | Unknown | | | 24 | | | 31 | | | 4 | | | 52 | | | 16~ | | | 29 | | | 2 | | | 2 | | |  |
| Isolate_10 | | 132~ | | | Unknown | | | 10 | | | 2 | | | 7 | | | 101 | | | 7 | | | 1 | | | 4 | | | 2~ | | |  |
| Isolate_11 | | 471 | | |  | | | 6 | | | 6 | | | 4 | | | 2 | | | 154 | | | 7 | | | 2 | | | 4 | | |  |
| Isolate_12 | | 139~ | | | Unknown | | | 136 | | | 176~ | | | 4 | | | 123~ | | | 74 | | | U | | | 160~ | | | 50 | | |  |
| Isolate_13 | | 809~ | | | Unknown | | | 10 | | | 3 | | | 4 | | | 83 | | | 41 | | | 1 | | | 4 | | | 2~ | | |  |
| Isolate_14 | | 88 | | |  | | | 32 | | | 47 | | | 4 | | | 10 | | | 16 | | | 7 | | | 4 | | | 5 | | |  |
| Isolate_15 | | 478 | | |  | | | 8 | | | 2 | | | 7 | | | 84 | | | 7 | | | 1 | | | 4 | | | 2 | | |  |
| Isolate_16 | | 2~ | | | Unknown | | | 8 | | | 2 | | | 7 | | | 146~ | | | 7 | | | 1 | | | 4 | | | 2 | | |  |
| Isolate_19 | | 94~ | | | Unknown | | | 72 | | | 52~ | | | 47 | | | 47~ | | | 28 | | | 37~ | | | 36 | | | 48 | | |  |
| Isolate_20 | | 338 | | |  | | | 24 | | | 3 | | | 3 | | | 26 | | | 16 | | | 108 | | | 4 | | | 2 | | |  |
| AgEc_271 | 21 | | |  | | | 7 | | | 33 | | | 18 | | | 2 | | | 5 | | | 28 | | | 2 | | | 2 | | |  |  |
| AgEc_18 | 2~ | | | Unknown | | | 8 | | | 2 | | | 7 | | | 146~ | | | 7 | | | 1 | | | 4 | | | 2~ | | |  |  |
| AgEc_76 | 403~ | | | Unknown | | | 18 | | | 8 | | | 112 | | | 11 | | | 8 | | | 12 | | | 13~ | | | 108 | | |  |  |
| AgEc_81 | | | 698~ | | | Unknown | | | 10 | | | 2 | | | 3 | | | 17~ | | | 18 | | | 1 | | | 4 | | | 2 | | |

Table S6. Distribution of virulence factor genes from different animal sources.

| Strains | Source | Phylogroup | ESBL + ve strains-  phenotype | Virulent factors |
| --- | --- | --- | --- | --- |
| 17 (1) | Beef | **B1** | **-** | entA, ygeH, cif, ycbU, idaACD, stgABCD, iagB, csgBDEFG, flgN, flgB, gspC, aec17,18,19, 24,25,26,29,31,32, ehaAB, gspFGJK, hlyE/clyA, flgDCEGHK, fliAIJNPGSY, ycbQRT, ppdD/hcpA, hofB/hcpB, flhCBD, cheABRWYZ, tar/cheM, motA, fimABCDEFGHI, escFJ, cesD2, espABD, sepL, escDR, cesFT, entCDEF, fepAG, ecpAB, gspLM, fepB, eae, aec28, rorf8, sepD, nleH1-1, G2-4, nleE, nleB1, espHR1, espL2, ibeBC, cfaABCD, ecpBDR, cesD, grlAR, csgC, espGH, tir, sepZ, csgA, upaG/ehaG, espL4 (123) |
| 30 (2) | Beef | **B1** | **-** | astA, flhBCDE, motA, cheABRYZW, tar/cheM, flgBCDGHJKN, fliAGJLNOSY, csgDEFG, hofC, ppdD, elfADG, csgB, ycbU, fimABCDIEFGH, entBC, ecpABCR, ybtAEPSTU, psn/fyuA, gspM, fepD, fimD, csgC, irp2, eprI, ibeBC, epaP, eprK, hlyE/clyA, aec31,32, csgA, espL4, ehaB, orgA, espR1, eaeH (81) |
| 37 (3) | Beef | **A** | **-** | astA, flhB, flgBCDEGHJKN, cheABRYWZ, flgN, csgG, stgD, stgABC, ycbU, gspBCDFG, csgAEF, tar/cheM, ycbQR, ycbQ, fliACDGLJMSPZ, flhCD, motA, gspMJ, ecpABDR, fimABCDFEGHI, hlyE/clyA, fepB, entD, gspL, fepAC, entACEF, eaeH, csgC, cfaB, ehaAB, upaG/ehaG, hcp, aec17,18,19,24,25,26,28,29,31,32, espR1, espL4, ibeBC, cfaCD, agn43, cdiB, traJ (103 VF) |
| 61 (4) | Dairy | **A** | **-** | astA, flgABCDFGHJKN, hofC, csgACDEFG, ycbU, flhB, ycbS, cheABWZ, cdt-IIIC, cdt-IIIB, cnf2, flk, fliACDEGQMLJPTRZ, flhD, flhC, motA, tar/cheM, cheRY, csgB, ycbQ, eprK, fimCBDIGHFE, shuV, fepABCD, entBCD, chuATSWY, yagX/ecpC, yagY/ecpB, gspCKLM, eprHI, epaP, eivCEIG, pkgA, gspD, hlyE/clyA, espY2,5, espX4, espL4, ibeB, eaeH, ecpAER, aec22,25, hlyC (100 VF) |
| 76(5) | Dairy | **A** | **+** | hlyE/clyA, csgABCDEF, stgBCD, ycbQU, eprI, pkgA, eivF, cdiB, ycbQ, upaG/ehaG, aec7,8,16,17,18,19,24, 25, 26, 30,31,32,flgABFGHJN, cdtC, cdt-IIIB, fliACDHJLMNQOR, ycbT, flhABCD, cheARWYZ, ycbR, fimABCDEFGHI, entABCD, chuATWUXS, ecpABD, ppdD, cfaABC, aec27/clpV, tar/cheM, gspLM, ehaB, fepD, espY4, ppdD, csgG, hofB, fepB, agn43, ibeB, stgA, flip, ecpR, traJ(105 VF) |
| 81(6) | Dairy | D | **+** | astA, tar/cheM, cheABRWY, flhBE, flgH, flgABCDFGH, ycbU, vgrG, gspI, hlyE/clyA, csgABCDEE, hofC, elfADG, motA, flk, fliACFEGJLMNOTZ, hcp, eivF, ecpAR, aec17, chuASTWX, fimABCDEFGH, ecpB, gspM, shuV, fepG, entA, gspK, ecpD, cfaB, aec15,19,24,25,26,28,29,32, cfaAC, ibeB, espR1, entC, ehaAB, fepB, stgC, flgDN, ibeC, flhA, entB, flhD, flgK, gspJ, flhC, ibeB (110 VF) |
| 95(7) | Dairy | D | **-** | astA, cheABRYZW, tar/cheM, flhBD, flgBCDEGHKN, csgACFG, stgD, motA, ycbQR, csgBDCE, flhC, fliAEGJLMPSYZ, stgB, ycbU, fimABCDEFGI, fepABC, gspL, ecpB, entACDEF, fimH, stgA, ecpR, ecpD, hlyE/clyA, gspG, ehaA, ecpA, upaG/ehaG, gspE, aec15,32, espX1, gspC, ibeBC, cfaBCD, etrA, gspM, gspI, espR1, ehaB, eaeH, flk (91 VF) |
| 96(8) | Dairy | **B1** | **-** | gspK, tar/cheM, cheABRYWZ, flhBE, flgBCDEFGHKN, hofC, ycbU, vgrG, flhC, csgCEFG, ppdD, elfADG, csgB, motA, fliAEGJLNOS, flhD, flk, aaiW, gspEG, fimABCDEFGH, entC, gspM, ecpE, ecpAB, fepAD, entB, cfaABC, eaeH, ibeB, gspH, hlyE/clyA, aec25,17,24,25,26,29,31,32, ibeC, aec27/clpV, csgA, ecpR, ehaB, gspJ, fimI, csgD, flgJ, espR1, espL4 (90 VF) |
| 198(9) | Swine | **B1** | **+** | gspG, flgBCDEFGHJKN, csgBCDFG, stgABC, ycbU, gspC, flhB, cheBYZ, cib, mrkABDF, flk, flip, fliALIGJTMZ, gspJLM, flhCD, motA, cheARW, tar/cheM, hlyE/clyA, cfaABD, eaeH, ecpABER, fimABCDEFGH, ibeB, fepABD, entACDEF, gspD, ycbQ, ibeC, espR1, ehaB, ycbS, upaG/ehaG, aec16, 17, 18, 19, 22, 24, 2526, 28, 29, 31,32, espL4, ppdD/hcpA, hofB/hcpB, espX1, ycbR, csgA, fimC, ehaA (105 VF) |
| 200(10) | Swine | D | **+** | fimF, fliAEGSZ, flhCD, motA, cheABYWZ, tar/cheM, fliJKLMO, csgABCDEFG, elfACD, cib, mrkABCF, flk, flgABDEHJFGN, fimBCDIEHFG, entC, gspJ, ecpAB, , fepD, entB, gspL, eaeH, ibeC, hofC, ycbU, ppdD, gspG, hlyE/clyA, gspE, ehaB, ibeB, ecpR, espL4, flhA, espR1 (77 VF) |
| 210(11) | Swine | **C** | **+** | entACE, flhBCD, motA, tar/cheM, cheBRWZ, flgBCDFEGHJKLN, csgABCDEFG, fliADERQPNLJHSYZ, ycbQ, mrkABCF, eaeH, ecpABCR, fimABCDEFGIH, fepABD, cfaABCD, stgABCD, ycbU, pkgA, gspL, hlyE/clyA, upaG/ehaG, aec15,31,32, espL4, ibeBC, tibA, ehaB, flk, espR1 (90 VF) |
| 214(12) | Swine | **C** | **+** | flgABCDEFGHJNK, csgABCDEFG, stgBD, ycbU, gspDL, hlyE/clyA, flhBCD, cheABWRYZ, ycbQRS, flk, flip, fliAGILJNSY, motA, tar/cheM, hcp, iutA, fimBCDEGHI, fepAD, entACD, ybtAEPSU, iucAB, agn43, yagW/ecpD, gspM, irp2, aec16,17,18,19,22,23,24,25, 26,28,31,32, espL1, ibeB, ecpR, cfaABD, ibeC, ecpABE, irp1, ehaAB, espL4 (106 VF) |
| 217(13) | Swine | D | **+** | AstA, tar/cheM, cheABWRYZ, flhBCD, flgBCDGHJLN, ycbU, eprHK, epaP, gspE, motA, csgACDEFG, hofB, ppdD, csgB, flk, flip, fliAGLJNSYZ, gspGH, eaeH, eltB, ecpBR, hlyC, hlyD, fimFG, virK, gspL, faeC, ecpC, msbB2, fepABD, entACDE, ecpA, faeEG, hlyE/clyA, ehaB, aec1517,18,19,23,25,26,28,29,32, faeE, espL4, ibeBC, faeHIJ, fimC, flgK, hlyA, hlyB, traJ, espR1 (VF 95) |
| 225(14) | Swine | **B1** | **+** | CheABRWYZ, flhCBD, flgBCDFGHEJKN, tar/cheM, csgFG, motA, ycbQR, csgABCDE, cib, fliAGJLNOZ, stgBD, fimBCDEFGHI, ecpR, entACEFD, ecpB, ecpA, fepABG, stgC, ecpD, cfaA, gspM, hlyE/clyA, ehaA, upaG/ehaG, aec17,18,19, 28,32 eaeH, gspL, espX1, ycbU, espR1, espL4, ibeC, stgA, cfaD, cfaB, flk, ibeB (VF 88) |
| 226(15) | Swine | **C** | **+** | gspM, fliAGILJOMRSZ, flhBCD, motA, cheABRWYZ, tar/cheM, csgBCDEFG, hofC, ppdD, elfACD, flk, flgBCDGHJKN, fimACDFFGHI, ecpC, ibeBC, ecpB, ecpAR, aec31,32, fepD, hlyE/clyA, epaP, eprI, entBC, eprK, ycbU, csgA, espL4, eaeH, ehaB, orgA, espR1 (73) |
| 267(16) | Poultry | D | **-** | upaG/ehaG, eivAF, pkgA, eprIJ, ycbQU, focY, stgABCD, flgHJBCDFNG, cheAWRYZ, tar/cheM, pulCDFlS, aec7,8,11,15, 16,17,18,19,24,25,26,28,29,30,31,32, hcp, ehaAB, cdiB, usp, gspGM, motA, clbABCDEGHIJLMOQR, cdt-IIIB, cdtC, cib, hma, elfAD, ppdD, csgABDEF, flhC, fliABCDEFGHIJLMOQKRSZ, , flk, fimADHGFCGEIBK, vat, tia, sfaABDEFGSX, ybtAEPTSU, irp2, papABC, chuASUTYW, sitB, entB, iroBCDEN, ecpBD, hlyABCD, virK, papEKJX, gspIK, kpsD, cfaABCD, ecpR, papD, csgA, gspFM, upaH, epaP, f17d-D, ibeBC, ipaH, espL4, ecpA, cdiA, espX1, fes, , hofB, entE, fepC, tibA, ecpE, gspL, gspI, fepG, traJ, gspD, orgA, csgC, upaG/ehaG, flhD, pulO, espR1 (VF 212) |
| 271(17) | Poultry | **E** | **-** | aec7,8,16,17,18,19,23,24,25,26,28,29,31,32, flgBCFGHK, ycbU, cheBRWY, eprI, hlyE/clyA, eivF, flhCD, csgABCDEF, elfACDG, fliASGHILJHEMNOZ, sitD, chuATSXUW, fimABCDEFGHI, ecpABER, gspLM, ibeBC eaeH, entABC, espL4, hofB, flgN, ppdD, traJ (VF 92) |
| 281(18) | Horse | **A** | **-** | gspLM, flgBCDGHKJN, csgABCDEFGYZ, tar/ cheM, cheABWZ, motA, flhACD, fliADESGIJNOR, elfADG, hofC/ hcpC, entBC, epD,ecpBD, ecpA, gspGI, aec26, 32, eprIJ, ycbFU, ibeBC, ecpR, espL4, hlyE, orgAB, gspE, flip, ehaB (VF 60) |
| 283(19) | Lamb | **E** | **-** | ycbQSV, flgBCDFGHJKLN, ppdD, hofC, csgABCDEFG, cheABRWYZ, flk, fliAEDFGLJNPSZYR, flhBCD, motA, tar/cheM, eprIK, chuAWSTX, fimBCDEFGHI, entBCD, ecpB, fepACD, yagX/ecpC, shuV, ecpR, ibeB, gspD, ecpA, aec14, 16, 17,18,19, 25,26,32, pkgA, hlyE/clyA, eivACFI, espY2, ibeC, usp, ehaB, hcp, astA (VF 95) |
| 287(20) | Sediment | **B1** | **-** | iucAD, cheABRYZ, tar/cheM, flhBCD, flgBCDEFGHKN, csgABCDEFG, stgBCD, motA, ycbQR, cdt-IIIC, cdt-IIIB, fliAGJLPSNYZ, f17d-C, fimBCDEFGHI, gspL, ecpBD, fepABC, entADEFC, stgA, ecpR, iutA, f17d-D, ycbV, etrA, gspCEGJM, hlyE/clyA, ehaAB, ecpA, upaG/ehaG, espX1, ibeBC, cfaABD, eaeH, espR1, hlyC (89).  TOTAL# of VF genes ≈ 1837 |

**Virulence factors (VF) distribution from different animal sources**

The presence of 1837 virulence factor genes were detected in the 20 isolates used in this study based on WGS (Table.S3 & 4). Poultry isolates contained higher numbers of VF genes than any other animal source used in this study, while horse isolates contained the lowest. There were no differences in the number of VF genes detected among the other four animal sources (beef, dairy, swine, lamb) and sediment. The most prevalent VF genes in the isolates were the *flg*, *fli*, *fim*, *che*, and the *csg* genes (Table S3 & 4).

Table S7. Twenty three reference *E. coli* genomes were chosen to represent a range of the species from the NCBI assembled genome database. Below with each genome's name within the SNP tree, along with the GenBank accession, refseq accession, and ST type (both the Achtman Schema #1 and Pasteur Schema #2).

| Genome | Genbank Accession | Refseq Accession | ST Type (Schema 1) | ST Type (Schema 2) |
| --- | --- | --- | --- | --- |
| Escherichia_coli_11128 | GCA_000010765.1 | GCF_000010765.1 | 16 | 480 |
| Escherichia_coli_12009 | GCA_000010745.1 | GCF_000010745.1 | 17 | 135 |
| Escherichia_coli_2011C-3493 | GCA_000299455.1 | GCF_000299455.1 | 678 | 290 |
| Escherichia_coli_400791 | GCA_001265435.1 | GCF_001265435.1 | 328~ | 279~ |
| Escherichia_coli_53638 | GCA_000167915.2 | GCF_000167915.2 | 6 | 372~ |
| Escherichia_coli_B185 | GCA_000163175.1 | GCF_000163175.1 | 3577 | 94~ |
| Escherichia_coli_B354 | GCA_000163195.1 | GCF_000163195.1 | 6961 | 99~ |
| Escherichia_coli_ETEC_H10407 | GCA_000210475.1 | GCF_000210475.1 | 48 | 132 |
| Escherichia_coli_H299 | GCA_000176695.2 | GCF_000176695.2 | 117 | 48 |
| Escherichia_coli_IAI39 | GCA_000026345.1 | GCF_000026345.1 | 62 | 254 |
| Escherichia_coli_K_12_substr_MG1655 | GCA_000005845.2 | GCF_000005845.2 | 10 | 262 |
| Escherichia_coli_KO11FL | GCA_000258025.1 | GCF_000258025.1 | 1079 | 360 |
| Escherichia_coli_KTE102 | GCA_000408585.1 | GCF_000408585.1 | 120 | 341 |
| Escherichia_coli_KTE186 | GCA_000408105.1 | GCF_000408105.1 | 452 | 142~ |
| Escherichia_coli_KTE33 | GCA_000398885.1 | GCF_000398885.1 | 5345 | 615~ |
| Escherichia_coli_KTE75 | GCA_000351685.1 | GCF_000351685.1 | 738 | 106~ |
| Escherichia_coli_KTE84 | GCA_000352465.1 | GCF_000352465.1 | 5394 | 579 |
| Escherichia_coli_M863 | GCA_000190955.1 | GCF_000190955.1 | 2715 | 670~ |
| Escherichia_coli_NRG_857C | GCA_000183345.1 | GCF_000183345.1 | 135 | 64 |
| Escherichia_coli_O157_H7_str_Sakai | GCA_000008865.2 | GCF_000008865.2 | 11 | 296 |
| Escherichia_coli_SE15 | GCA_000010485.1 | GCF_000010485.1 | 131 | 506 |
| Escherichia_coli_UMEA_3318-1 | GCA_000461235.1 | GCF_000461235.1 | 10 | 466 |
| Escherichia_coli_UMNK88 | GCA_000212715.2 | GCF_000212715.2 | 100 | 809~ |

S.2. . Phylo-grouping

Quadruplex PCR assay (Fig. 1A and Fig. S1B) showed that the most prevalent phylogenetic groups were A and B1 (A: 92/300, 30%; B1: 87/300, 29%), followed by D and E, each with 21 isolates. Furthermore, and within each animal source, 14.28, 19.00, 42.8 and 8.8% for phylo-group A, were from beef (n=7), dairy (n=20), swine (n=29), and poultry (n=7), respectively. Also, 19.7, 20.9, 29.1, and 19.8% for phylo-group B1 were from beef (n=5), dairy (n=18), swine (n=43), and poultry (n=3), respectively. The rest of the animal sources and environmental samples showed lower distributions of phylo-groups, although these samples had fewer numbers of isolates. Phylogenetic group A has been shown to be highly associated with commensal strains [1], and in this study it represents about 30.7% of the isolates while isolates from group B1 represent about 29% of the population. Clinical isolates from phylo-group A have been associated with urinary tract infections [2-3], and some B1 clinical strains are also known to cause urinary tract infections. About 10% of the isolates tested in this study belong to phylo-group B2, and previous studies have shown that extraintestinal pathogenic E. coli strains belong to phylogenetic groups B2 and D [4, 5].

**Fig S1A.** Phylo-group by quadruplex PCR assay. The most prevalent phylo-groups were A and B1.

Fig. S1B. Phylo-group by quadruplex PCR assay. The most prevalent phylo-groups were A and B1, then followed by D and E. Within each animal source, 14.28, 19.00, 42.8 and 8.8% for phylo-group A, were from beef, dairy, swine, and poultry, respectively. Also, 19.7, 20.9, 29.1, and 19.8% for phylo-group B1 were from beef, dairy, swine, and poultry, respectively. The rest of the animal sources and environmental samples showed lower distributions of phylo-groups, although these samples had fewer numbers of isolates. The 12 states that isolates were collected are California (CA), Connecticut (CT), North Carolina (NC), North Dakota (ND), Wisconsin (WI), Idaho (ID), Nabraska (NE), Washington (WA), Giorgia (GA), Kentucky (KY), South Carolina (SC),  Illinois (IL).

S.3. Susceptibilities of Isolates against 18 Antibiotics

The 300 *E. coli* isolates were screened for susceptibility against eighteen antimicrobials. Of the 300 isolates, 59.7% were resistant to sulfisoxazole, 49.3% to tetracycline, 32.3% to cephalothin, 22.3% to ampicillin, 20% to streptomycin, 16% to ticarcillin, and the remaining 12 antimicrobials carried less than 10% resistance (Fig. S2). Of the 300 *E. coli* isolates, 116 isolates were identified as multidrug resistant (MDR), i.e., resistance to three or more antimicrobial drug classes. The antimicrobials associated with most MDR were tetracycline with 94 isolates (31.3%), sulfisoxazole with 88 isolates (29.3%), ampicillin with 64 isolates (21.3%), cephalothin with 60 isolates (20.0%), streptomycin with 46 isolates (15.3%), and ticarcillin with 44 isolates (14.7%). Detailed examination of isolates from the different animal sources showed that 11% (5/38)) of isolates from beef, 39.8% (39/98) of isolates from dairy, 56% (56/100) of isolates from swine, and 35% (13/37) of isolates from poultry were MDR. One isolate, each from fish, sediment, and river was MDR. Interestingly, none of the isolates from horse and lamb were MDR. Furthermore, most of the MDR belonged to phylo-group A: 12/39 in dairy, 26/56 in swine, and 3/13 in poultry. These data suggested swine was the animal source that harbored the highest percent MDR isolates from this study, and phylo-group A with the highest MDR isolates in swine, dairy, and poultry. AR E. coli is common and widespread in agricultural environments [65]. Most animals excrete antibiotic resistance genes into the environment [1], and the transfer of these genes to other bacteria are of great concern to human health [66].

Since 2010, many studies have determined antibiotic resistance in animal production environments [9-15]. However, in many watersheds, non-agricultural environments may produce the same or higher AR bacteria [16-21]. Furthermore, the transport of pathogens carrying MDR associated with integrons or mobile DNA elements such as plasmids and transposons from animal feces to the environment has been well documented [22, 23]. Consequently, pathogens associated with animal feces and with increased resistances may be transported from animal manure into rivers and other environmental components [24]. For example, a study was conducted to determine the impact of nontherapeutic use of antibiotics on swine manure-impacted water sources, surface water and groundwater situated up and down the gradient from a swine facility [25]. Higher numbers of erythromycin- and tetracycline-resistant enterococci were detected in down-gradient surface waters. The authors suggested that water contaminated with swine manure could contribute to the spread of antibiotic resistance in the environment. Similarly, we also observed elevated levels of resistance to tetracycline and streptomycin from swine manure and effluent from swine through a constructed wetland [65]. In the current study, the most frequently detected resistant phenotypes were to sulfisoxazole, tetracycline, cephalothin, and ampicillin, which were detected in all ESBL isolates from swine.

**Figure S2.** Percent susceptibility of 300 *E. coli* isolates against18 antibiotics.


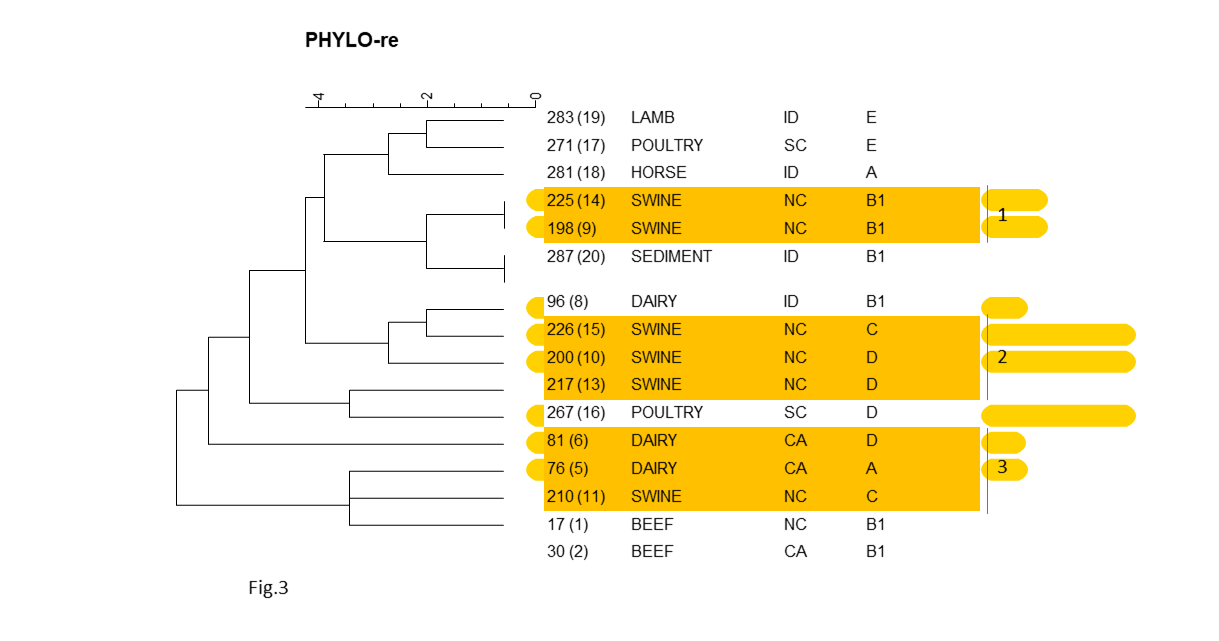


214 (12) swine NC B1

**Figure S3.** Detection of ESBL *E. coli* phenotype by double synergy test. All 300 *E. coli* isolates were screened for ESBL production on TBX media supplemented with 4 mg/L cefotaxime (TBX-CTX) for detection of ESBL *E. coli*. A total of nine isolates were positive for ESBL production, and eleven additional isolates from other animal and environmental sources were included for whole genome sequencing. Data marked in color are the ESBL positive isolates (seven from swine and two from dairy). In most instances, the genes coding for the observed phenotypes were identified using WGS. Isolates 12 is from swine and that makes 7 isolates.

**References**

1. Moreno, E., Johnson, J. R., P´erez, T., Prats, G., Kuskowski, M. A., Andreu, A. Structure and urovirulence characteristics of the fecal *Escherichia coli* population among healthy women. *Microbes. Infect.* **2009**, 11, 274–280.
2. Moreno, E., Andreu, A., Pigrau, C., Kuskowski, M. A., Johnson, J. R., Prats, G. Relationship between *Escherichia coli* strains causing acute cystitis in women and the fecal *E. coli* population of the host,” *J. Clin. Microbiol.* 2008, 46: 2529–2534.
3. Rendon, M. A., Saldana, Z., Erdemet, A. L., Monteiro-Neto, V., Vazquez, A., Kaper, J. B., Puente, J. L., Giro, J. A. Commensal and pathogenic *Escherichia coli* use a common pilus adherence factor for epithelial cell colonization,” *PNAS*. **2007**, 104: 10637–10642.
4. Johnson, J. R., Stell, A. L. Extended virulence genotypes of *Escherichia coli* strains from patients with urosepsis in relation to phylogeny and host compromise. *J. Infect. Dis.* **2000**, 181, 261–272.
5. Pompilio, A., Crocetta, V., Savini, V., Petrelli, D., Di Nicola, M., Bucco, S. et al. Phylogenetic relationships, biofilm formation, motility, antibiotic resistance and extended virulence genotypes among Escherichia coli strains from women with community-onset primitive acute pyelonephritis. *PLoS ONE* **2018**, 13(5): e0196260. https://doi.org/10.1371/journal.pone.0196260.
6. Ibekwe, A. M., Murinda, S. E, DebRoy, C., Reddy, G. B. Potential pathogens,

antimicrobial patterns, and genotypic diversity of Escherichia coli isolates in constructed wetlands treating swine wastewater. *FEMS Microbiol. Ecol*. 2016, doi: 10.1093/femsec/fiw006.

1. Durso, L. M., Miller, D. N., Wienhold, B. J. Distribution and quantification of antibiotic resistant genes and bacteria across agricultural and non-agricultural metagenomes. *PLoS ONE*.**2012**, 7: e48325. doi:10.1371/journal.pone.0048325.
2. Normark, B. H., Normark, S. Evolution and spread of antibiotic resistance. J. Intern. Med. 2002, 252: 91–106.
3. Agga, G. E., Arthur, T. M., Durso, L. M., Harhay, D. M., Schmidt, J. W. Antimicrobial-Resistant Bacterial Populations and Antimicrobial Resistance Genes Obtained from Environments Impacted by Livestock and Municipal Waste. *PLoS ONE* **2015**, 10(7): e0132586. https://doi.org/10.1371/journal.pone.0132586
4. Agga, G. E., Scott, H. M, Amachawadi, R. G., Nagaraja, T. G., Vinasco, J. et al. Effects of chlortetracycline and copper supplementation on antimicrobial resistance of fecal Escherichia coli from weaned pigs. *Prev*. *Vet*. *Med*. **2014,** 114: 231–246. pmid:24655578.
5. Brichta-Harhay, D. M., Arthur, T. M., Bosilevac, J. M., Kalchayanand, N., Shackelford, S. D. et al. Diversity of multidrug-resistant *Salmonella enterica* strains associated with cattle at harvest in the United States. *Appl. Environ. Microbiol*. **2011**, 77, 1783–1796.
6. Brooks, J. P, Adeli, A., McLaughlin, M. R. Microbial ecology, bacterial pathogens, and antibiotic resistant genes in swine manure wastewater as influenced by three swine management systems. *Water Res* **2014**, 57C: 96–103.
7. Dungan, R. S., Klein, M., Leytem, A. B. Quantification of bacterial indicators and zoonotic pathogens in dairy wastewater ponds. *Appl. Environ. Microbiol*.  **2012**, 78, 8089-8095; DOI: 10.1128/AEM.02470-12.
8. Frye, J. G., Jackson, C. R. Genetic mechanisms of antimicrobial resistance identified in Salmonella enterica, *Escherichia coli*, and *Enteroccocus* spp. isolated from U.S. food animals. *Front. Microbiol*. **2013**, 4, 135. doi: 10.3389/fmicb.2013.00135
9. Haley, C. A., Dargatz, D. A., Bush, E. J., Erdman, M. M., Fedorka-Cray, P. J.

*Salmonella* prevalence and antimicrobial susceptibility from the National Animal Health Monitoring System Swine 2000 and 2006 studies. *J. Food Prot*. **2012**, 75, 428–436.

1. Berendonk, T. U., Manaia, C. M., Merlin, C., Fatta-Kassinos, D., Cytryn, E., Walsh, F., et al. Tackling antibiotic resistance: the environmental framework. *Nat Rev Microbiol*. **2015**, 13, 310-7. doi: 10.1038/nrmicro3439.
2. McKinney, C. W., Loftin, K. A., Davis, J. G., Meyer, M. T., Pruden, A. tet and *sul* antibiotic resistance genes in livestock lagoons of various operation type, configuration, and antibiotic occurrence. *Environ. Sci. Technol*. **2012**, 44, 6102−6109.
3. Pruden, A., Pei, R., Storteboom, H. N., Carlson, K. H. Antibiotic resistance genes as emerging contaminants: studies in northern Colorado. *Environ. Sci. Technol*. **2006**, 40, 7445−7450.
4. Pruden, A., Arabi, M., Storteboom, H. N. Correlation between upstream human activities and riverine antibiotic resistance Genes. *Environ. Sci. Technol*. **2012**, 46, 6102−6109.
5. Storteboom, H. N., Arabi, M., Davis, J. G., Crimi, B., Pruden, A. Tracking antibiotic resistance genes in the South Platte River basin using molecular signatures of urban, agricultural, and pristine sources. *Environ. Sci. Technol*. **2010**, 44, 7397−7404.
6. Storteboom, H. N., Arabi, M., Davis, J. G., Crimi, B., Pruden, A. Identification of antibiotic resistance gene molecular signatures suitable as tracers of pristine river, urban, and agricultural sources. *Environ. Sci. Technol.* **2010**, 44, 1947−1953.
7. Aarestrup, F. M., Kruse, H., Tast, E., Hammerum, A. M., Jensen, L. B. Associations between the use of antimicrobial agents for growth promotion and the occurrence of resistance among Enterococcus faecium from broilers and pigs in Denmark, Finland, and Norway. *Microbial Drug Resist*. **2000**, 6, 63–70.
8. Murinda, S. E., Ebner, P. D., Nguyen, L. T., Mathew, A. G., Oliver, S. P. Antimicrobial resistance and class 1 integrons in pathogenic *Escherichia coli* from dairy farms. *Foodborne Path. Dis*. **2005**, 2, 348-352.
9. Baquero, F., Martınez, J-L. Canton R. Antibiotics and antibiotic resistance in water environments. *Current Opinion Biotech*. **2008**, 9, 260–265.
10. Sapkota, A. R., Curriero, F. C., Gibson, K. E., Schwab, K. J. Antibiotic-resistant enterococci and fecal indicators in surface water and groundwater impacted by a concentrated swine feeding operation. *Environ. Health Perspectives*. **2007**, 115, 1040–1045.
