# Supplementary figures and images for: Diversity of Plasmids and Genes Encoding Resistance to Extended-Spectrum β-Lactamase in Escherichia coli from Different Animal Sources
Source: Microorganisms. 2021 May 13;9(5):1057. doi: 10.3390/microorganisms9051057 (PMC8153348; doi:10.3390/microorganisms9051057)

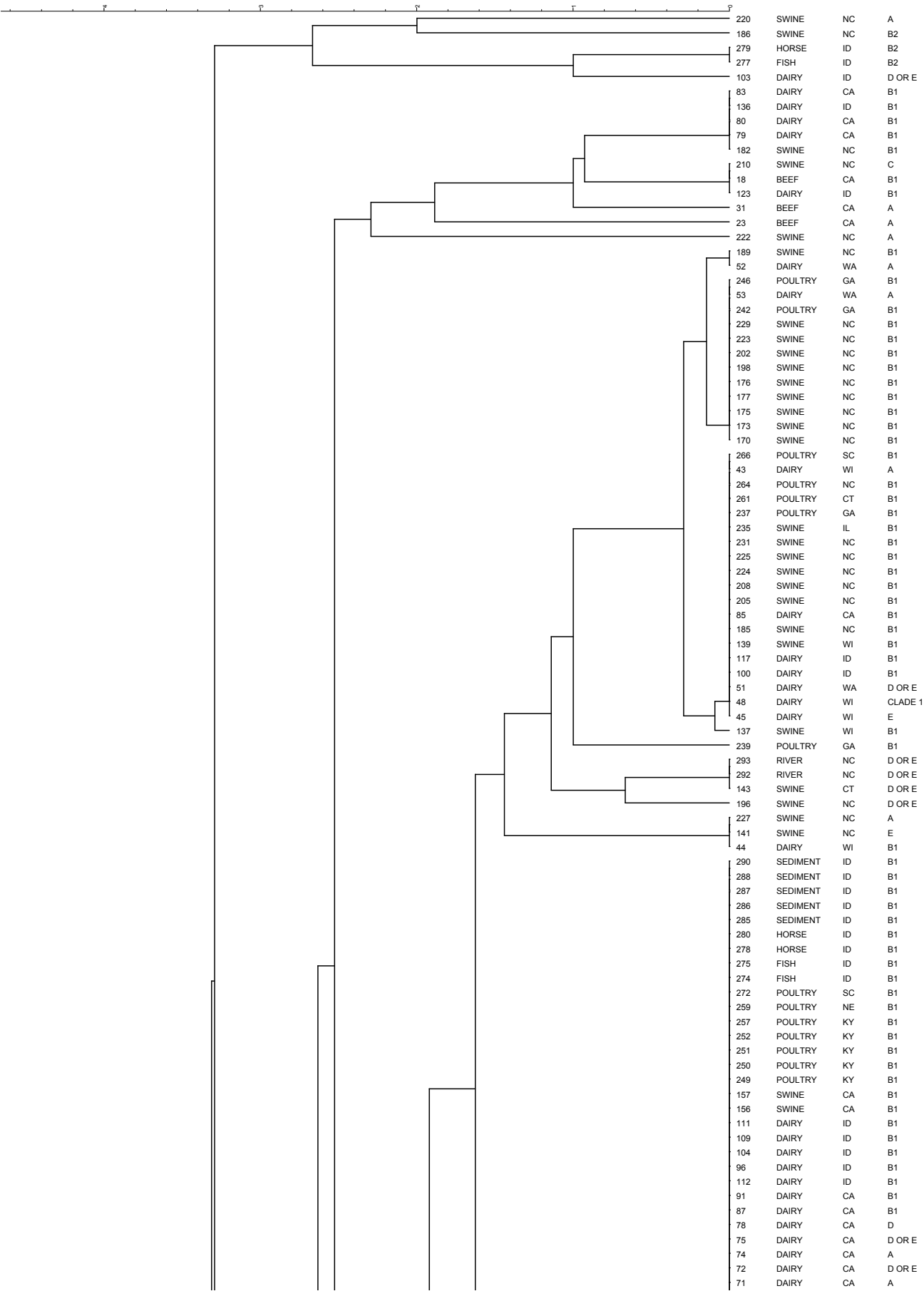

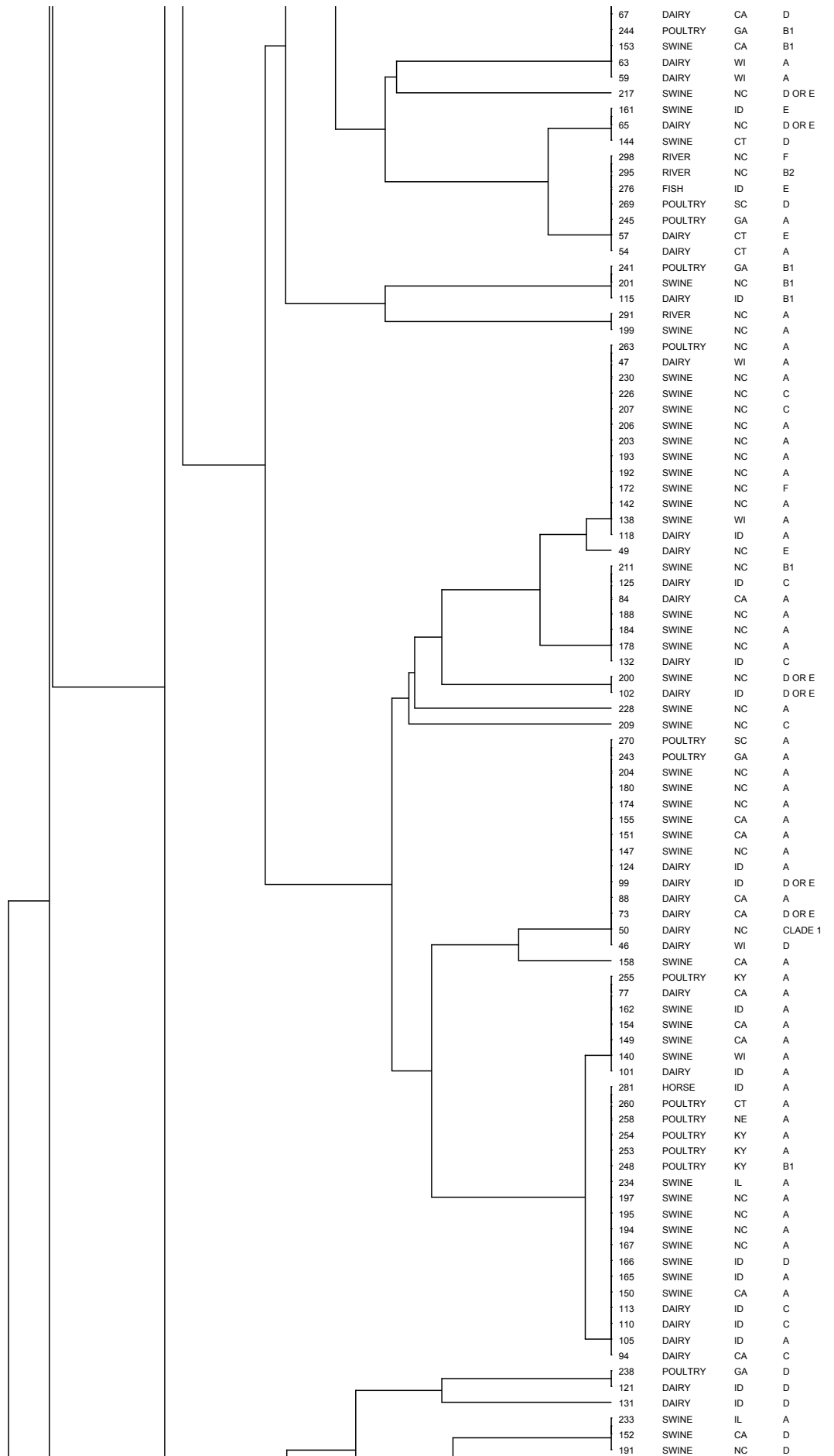

Supplement: Supplementary file 1 [file microorganisms-09-01057-s001.zip › Microbe-Fig.S1-PHYLO PHYLOGENETIC TREE (WHOLE).pdf]
